# Triadic Mn–ZnS/MOF/MIP Fluorescent Sensor for Highly Sensitive and Selective Sulfathiazole Detection

**DOI:** 10.3390/bios16090459

**Published:** 2026-08-24

**Authors:** Fatih Pekdemir, İzzet Koçak

**Affiliations:** 1 Department of Chemistry, Faculty of Science, Zonguldak Bülent Ecevit University, Zonguldak 67100, Turkey; 2 Faculty of Pharmacy, Zonguldak Bülent Ecevit University, Zonguldak 67800, Turkey; izzetkocak@beun.edu.tr

**Keywords:** Mn-ZnS QD, MIP, MOF-5, sulfathiazole

## Abstract

Sulfathiazole (STH) pollution has been increasing, with a strong need for a sensitive and selective analysis method. In this paper, a three-component fluorescent sensing platform with Mn^2+^-doped ZnS quantum dots in a metal–organic framework coated with a surface molecularly imprinted polymer (Mn-ZnS-MOF-MIP) for selective sensing of sulfathiazole is proposed. This synergistic sensor platform combines Mn-ZnS-insensitive emission, MOF-assisted analyte enrichment, and imprinting-based molecular recognition. The sensing platform displays a characteristic turn-off fluorescence with a dominant static quenching mechanism. Under optimal conditions, a broad sensing range with a low detection limit of 13.75 nM can be obtained. Outstanding selectivity among similar sulfonamides, biomolecules, and metal ions were achieved with high reproducibility, stability, and reusability. This sensing platform was shown to analyze sulfathiazole accurately in aqueous samples and blood serum with high recoveries, within the range of 90.00–104.24%, compared to high-performance liquid chromatography methods.

## 1. Introduction

Sulfathiazole (STH), a sulfonamide antibiotic, is widely used as a veterinary drug and fishery agent because of its broad-spectrum antibacterial activity [1,2]. However, the widespread use of STH leads to pervasive environmental contamination through farm runoff and improper waste treatment [3,4]. STH remains present in the soil and water environment, significantly increasing the prevalence of antimicrobial resistance genes and triggering serious ecotoxicological concerns for aquatic organisms and human health through the food chain [2,5]. For this reason, the detection of STH with high accuracy, reliability, and sensitivity in complex samples, including biological fluids, foods, and aqueous media, becomes a priority for monitoring contamination, ecotoxicological analysis, regulatory control, or protecting public health [3,5]. Traditional analysis may not provide effective monitoring results for on-site or rapid field analysis; therefore, reliable sensing systems must be designed [1,4].

Conventionally, analysis of sulfonamides, including STH, has been performed using analytical tools such as high-performance liquid chromatography interfaced with mass spectrometry (HPLC-MS/MS) and enzyme-linked immunosorbent assay (ELISA) [6,7,8]. Although HPLC-MS/MS analysis is extremely sensitive and specific, it has significant drawbacks, including high operating costs and complex sample preparation, which demand highly specialized personnel and sophisticated equipment, making it highly unsuitable for high-throughput analysis or point-of-care testing [9,10,11]. ELISA, although simpler and easily multiplexed, has potential drawbacks, including issues of cross-reactivity between similar sulfonamide compounds that could cause false-positive results, and reagents that can have poor stability during exposures to high temperatures and varying pH [12,13]. Also considered have been electrochemical methods, which could have challenges of electrode poisoning and coexistence electroactive materials in real samples that cause unwanted interferences [14,15]. All of these challenges point to an important and critical need for innovative approaches that ideally integrate high selectivity, sensitivity, and simplicity and robustness into a functional analytical tool, making fluorescence sensing an important and attractive alternative for particular applications that pose challenges based on speed, cost, complexity of analysis, and analytical potential during miniaturization and point-of-time analysis and testing [16,17].

In this regard, quantum dots (QDs) have been of intensive research interest as superior fluorophores to conventional organic dyes and fluorescent proteins for many applications [18,19]. Their unique size-tunable photoluminescence, high quantum yields, broad absorption spectra, excellent photostability, and resistance to chemical degradation make them ideal candidates for constructing high-performance fluorescent sensors and labels [20]. Of the many kinds of QDs, Mn-ZnS QDs represent an important advance owing to their particular photophysical properties [21,22]. Doping Mn^2+^ ions in ZnS QDs opens new emission channels, resulting in a long-lived orange-red emissive (~590 nm) that is largely insensitive to nanocrystal size variations and characterized by a large Stokes shift [21,22]. This intrinsic dopant emission is less sensitive to photobleaching and environmental interference than the intrinsic band-edge blue emission of undoped ZnS or the surface state-dependent emission of Cd-based QDs [21,23]. The deliberate switch to Mn-ZnS QDs effectively minimizes several troubles in fluorescence sensing, such as the autofluorescence from biological samples and scattering from complex matrices, thus enabling a marked improvement in the signal-to-noise ratio, assay reliability, and time-gated detection to eliminate short-lived background fluorescence [20,21,24].

Running in parallel to the advances in the properties of QDs is the development of MOFs that has wrought a renaissance in the last two decades by showing expansive potential in catalysis, gas adsorption, separation, drug delivery, and chemical sensing [25,26,27,28]. A class of crystalline porous solids, MOFs consist of metal ions/clusters serving as nodes that are coordinated to multifunctional organic linkers, more commonly referred to as struts [26,29]. Their enormous potential in science stems from their unique property of designability on the molecular scale, facilitating the deliberate manipulation of chemical properties and porous structure, as well as other structural features, as required for a given application [30]. When used in fluorescence-based sensing assays, the applications of MOFs are multifaceted, working as protective hosts/matrix for the confinement of QD nanoparticles, acting as highly adept concentration matrices due to their high porosity, interacting in a target-specific manner, and thus acting as highly sensitive pre-concentration tools, since the resultant locally concentrated target analyte would significantly alter the fluorescence response—whether manifested as signal enhancement or quenching, depending on the specific photophysical relationship between the QD donor and target acceptor—finally acting as fluorescence modulation tools via Förster Resonance Energy Transfer (FRET) and/or photoinduced electron transfer (PET) principles [31,32]. Key issues with independently operated QD-based sensing, such as aggregation-induced quenching, poor stability in challenging environments, and nonspecific analyte interactions that impair selectivity, are directly addressed by this strategic integration [31,32,33,34].

However, to provide molecular selectivity—a phenomenon that is rather a common drawback of conventional QD sensors or MOF sensors based on passive adsorption or nonspecific interactions—molecularly imprinted polymers (MIPs) are incorporated as synthetic polymeric receptors possessing specifically designed three-dimensional polymeric sites that match the size, shape, and functional groups of a target molecular entity (template) according to the lock-and-key model for biological antibody–antigen interactions but with significantly improved chemical and physical resistance [35,36]. They are applied extensively as solid-phase extraction materials, chromatographic separations, drug delivery carriers, and chemical sensors [37]. The combination of MIPs with QDs within fluorescence sensors offers a type of “plastic antibodies” or “biomimetic sensors” where the binding process at the imprinted sites directly affects the fluorescence intensity, spectra, or decay-time properties of the QD with remarkably high selectivity from the synthetic recognition sites [38,39]. However, conventional bulk polymerization approaches for preforming MIP/QD combinations are known to suffer from problems such as incomplete removal of the templates, the formation of deeply buried imprinted sites that are inaccessible within the polymeric matrices with limited mass-transfer rates, and nonuniform distributions of imprinted sites [36,40].

This research outlines a triple-component integration approach to systematically address these long-standing limitations. A novel core–shell–satellite nanoparticle architecture is proposed by first incorporating the luminescent Mn-ZnS QDs in a porous and stabilizing MOF host material, which is then coated by a thin and highly accessible MIP layer synthesized through surface imprinting methods. It is suggested that such an architecture would facilitate a range of advantageous outcomes: (i) The MOF host would physically serve as a rigid and porous scaffold for protecting the aggregated QD nanoparticles and providing a synergistic molecular concentration of the target STH molecules in its nano-pores, hence improving the sensitivity by locally increasing the analyte concentrations. (ii) A thin and conformal MIP layer would be grown in situ around the MOF nano-tablet substrate in a highly customizable manner and with high specificity by providing universal and high-affinity binding pockets synthesized around the MOF-protected QD nano-particles for optimal signal transduction. (iii) Such a chemically inorganic–organic hybrid architecture would possess enhanced inherent chemical and photonic thermostability and robust resistance to possible degradations compared to its constituent parts, and hence, it is suitable for storage and utilization.

The aim of this research study, therefore, is to generate, systematically characterize, and critically test this new Mn-ZnS-MOF-MIP nanocomposite material for its potential as a turn-off fluorescent sensor for STH. These capabilities of this sensor material will specifically be systematically interrogated for its sensitivity, selectivity, speed of response, and ability for reuse, as well as real-world usability for water and bodily fluid samples spiked with this antibiotic sulfonamide. However, it is the crucial triadic combination approach here of a doped QD (for enhanced and matrix-independent emission), an MOF (for preconcentration of the target species and for subsequent protection of the fluorophore against degradation), and an MIP (for biorecognition specificity) into an optimal nanostructured form that fundamentally differentiates this contribution in the literature from that found in previous research papers, which might involve either binary nano-composites such as MOF-QD-based fluorescent sensors or MIP-QD-based sensor constructs. Such a comprehensive design is expected to confer the sensor with a remarkably favorable combination of durability, selectivity, sensitivity, and rapid response, thus establishing a novel benchmark for the fluorescence-based detection of sulfonamide pollutants and, by extension, other emerging pollutants as well.

## 2. Materials and Methods

Na_2_HPO_4_, NaH_2_PO_4_, NaCl, and ascorbic acid (AA) were purchased from Merck Chemicals (Darmstadt, Germany). The chemicals employed were azobisisobutyronitrile (AIBN), chelidamic acid, zinc acetate, manganese acetate, polyethylene glycol, ethylene glycol dimethacrylate (EGDMA), glucose, fructose, maltose, sucrose, dopamine, terephthalic acid, chloroform, lactose, NaOH, L-cysteine, sulfadiazine (SDZ), sulfaguanidine (SGA), sulfamerazine (SMR), sulfanamide (SA), sulfathiazole (STH), acetonitrile, DMF, sodium sulfide, and metal chlorides, all obtained from Sigma Aldrich (St Louis, MO, USA). Ethanol, methanol, glycine, uric acid, and dopamine were also purchased from Fisher Scientific (Pittsburgh, PA, USA). Thiophene acetic acid (TAA) was also obtained from Chem Bio (İstanbul, Turkey).

All fluorescence measurements of composite materials were performed by an LS-55 Fluorescence Spectrometer manufactured by the PerkinElmer company (Shelton, CT, USA), using a 1 cm pathlength quartz cuvette, with excitation and emission slit widths both set to 5 nm. Firstly, full-spectrum scan measurements were performed to identify suitable excitation wavelengths for characterizing fluorescence properties of composite materials. As a consequence of scanning experiments, an excitation wavelength of 290 nm was chosen, and emission spectra were recorded in the range of 500–650 nm; a representative full emission spectrum (300–700 nm) confirming this choice is provided in Figure S19. Finally, working solutions were prepared by dispersing an accurate weight of Mn-ZnS-MOF-MIP or Mn-ZnS-MOF-NIP into phosphate-buffered saline aqueous solution with ultrasonic treatment in order to achieve homogeneity in each working solution, with a final volume of 2 mg/5 mL, followed by the addition of different concentrations of STH—prepared from 1 mM stock solution in phosphate buffer solution (pH 7)—and the corresponding fluorescence intensities were measured.

Scanning electron microscopic studies were used to examine the morphological changes by employing a scanning electron microscope, TESCAN MAIA3 XMU (Brno, Czech Republic). An X-ray diffractogram method, employing a Rigaku Smart Lab (Tokyo, Japan), was used for understanding the crystalline structure of the regarding materials. Adsorption/desorption analysis was carried out by employing an ASAP2020 instrument (Micromeritics, Norcross, GA, USA). Fourier-transform infra-red spectroscopy (FTIR) measurements were performed with KBr pellets by employing an FTIR Spectra 100 (PerkinElmer, Shelton, CT, USA) within a wave number range of 4000–500 cm^−1^. HPLC measurements were also carried out using Shimadzu SPD-20AV LC system (Kyoto, Japan), along with a UV detector. UV–Vis absorption spectra were obtained using a UV/Vis Carry 100Bio spectrophotometer (Palo Alto, CA, USA).

### 2.1. The Synthesis of MOF5

The synthesis of MOF5 was based on a literature method [41]. Solution A was prepared by dissolving 3.40 g of Zn (OAc)_2_·2H_2_O, amounting to 15.4 mmol, in 100 mL of DMF. Solution B was prepared by dissolving 1.00 g of terephthalic acid (6.0 mmol) and 1.7 mL of triethylamine in 80 mL of DMF. At the room temperature, solution B was carefully added to solution A while the latter was stirred at 400 rpm for 3 h. The white precipitate was collected via centrifugation at 5000 rpm for 5 min. Subsequently, three washing steps were conducted using DMF, each of which was accompanied by a centrifugation step at 5000 rpm for 3 min. The precipitate was then washed three times with 15 mL of HPLC-grade CHCl_3_, and each wash was accompanied by a 24 h settling period. The final product was then dried under vacuum at 60 °C, yielding 1.65 g of MOF5.

### 2.2. The Synthesis of Mn-ZnS QD

The synthesis of Mn-ZnS QD was carried out based on a literature method [42], with slight modifications. Subsequently, 2.62 g (12.0 mmol) of Zn(CH_3_COO)_2_·2H_2_O and 0.39 g (1.6 mmol) of Mn(CH_3_COO)_2_·4H_2_O were dissolved in 30 mL of ultrapure water. The solution obtained was stirred at 1000 rpm in the presence of a nitrogen atmosphere. Then, a solution of Na_2_S·9H_2_O (2.88 g 12.0 mmol) in 30 mL of ultrapure water was added to the solution, while it was stirred continuously at 1000 rpm over 15 min. After that, 0.35 g of PEG-1500 dissolved in 20 mL of ultrapure water was added to the solution at 1000 rpm over 5–6 min. After that, the reaction system was stirred at 1000 rpm for 12 h at room temperature. The obtained white suspension was then subjected to centrifugation at 5000 rpm for 5 min at room temperature. The obtained precipitate was washed three times with 10 mL of ultrapure water. The obtained precipitate was again washed three times with absolute ethanol. Subsequently, the obtained precipitate was dried in the presence of a vacuum at room temperature in the dark. The precipitate obtained was stored at 4 °C. The obtained precipitate weighed 1.30 g, yielding 86%.

### 2.3. The Synthesis of MOF5–Mn-ZnS

In a typical experiment, 100 mg of MOF-5 and 200 mg of Mn-doped ZnS (Mn-ZnS) were mixed and ground with an agate mortar and pestle. The mixture was then suspended in 30 mL of absolute ethanol, sonicated at 37 kHz for 1 h in a sealed 50 mL round-bottomed flask while maintaining the temperature at 30–35 °C, and then magnetically stirred at 1000 rpm for 24 h under room-temperature conditions. The resulting solid product was collected via centrifugation (5000 rpm, 5 min) and dried in a vacuum at room temperature in the dark.

### 2.4. The Synthesis of MOF5–Mn-ZnS–MIP

**Scheme 1:**
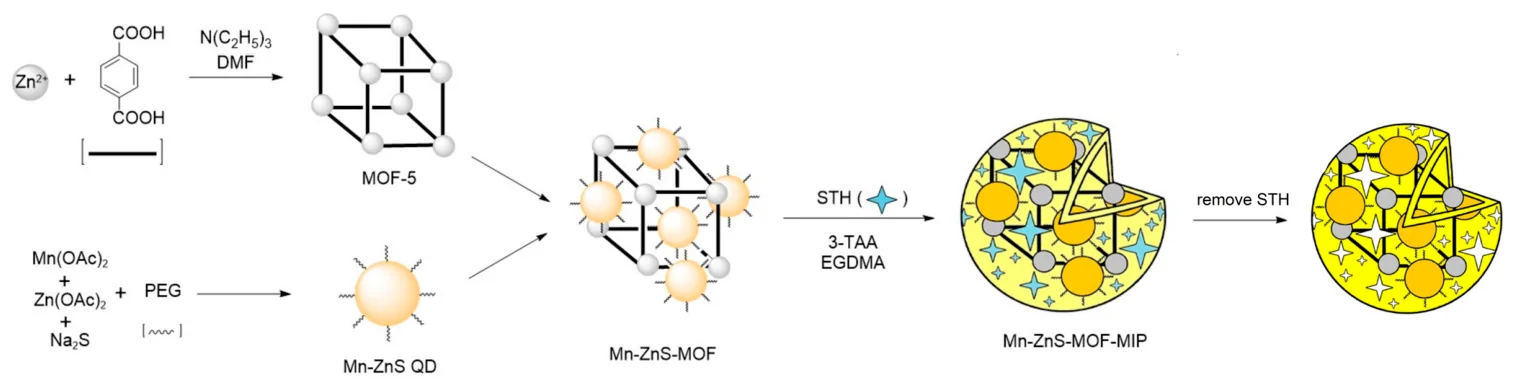
Schematic representation for the development of Mn-ZnS-MOF-MIP-based fluorescence sensor.

In a typical experiment, 50 mg of MOF-5–MnZnS was dispersed into 5 mL of acetonitrile in a 25 mL round-bottom flask under magnetic stirring. Subsequently, 25 mg (1 equiv., 0.1 mmol) of ST and 14 mg (1 equiv., 0.1 mmol) of 3-TAA were separately added, and pre-complex formation was allowed to stir at room temperature for 15 min. Thereafter, 300 µL of ethylene glycol dimethacrylate (EGDMA) was added dropwise, followed by the addition of 20 mg of 2,2′-azobis(2-methylpropionitrile) (AIBN) into the system. Sealing of the flask under nitrogen gas was followed by the stirring of the system at 500 rpm within the water bath at 50 °C for 1 h and subsequent stirring for 12 h at room temperature [43,44,45]. The white-colored compound formed was sufficiently washed using ethyl ether before being dried using the desiccator apparatus. Then, 200 mg of MOF-5–MnZnS–MIP was finely ground using an agate mortar and pestle and then placed in a Soxhlet extractor. Template removal was conducted via Soxhlet extraction using a methanol/acetic acid mixture (4:1, *v*/*v*) for 10 siphon cycles [44,45]. The obtained solid after the extraction was washed with hot methanol and subsequently with diethyl ether and dried in a desiccator. To confirm that the sensor’s enhanced response arises specifically from the molecularly imprinted recognition sites rather than from nonspecific interactions with the polymer matrix, a non-imprinted polymer (NIP) was also synthesized under identical conditions, but in the absence of the template molecule (STZ). Because the NIP lacks the shape- and functionality-complementary cavities generated during the imprinting process, it exhibits only weak, non-selective interactions with the target analyte. Comparing the sensor’s response toward the MIP and NIP therefore serves as an essential control, distinguishing selective molecular recognition from nonspecific adsorption as the basis of the sensing mechanism. The details of all the steps involved in the production of the fluorescence sensor are also given in Scheme 1.

### 2.5. Kinetics and Isotherms of STH Adsorption Experiments

To investigate the interaction of STH and Mn-ZnS-MOF-MIP, adsorption kinetics and adsorption isotherm experiments were conducted at room temperature. For kinetics studies, 1 mg of Mn-ZnS-MOF-MIP or Mn-ZnS-MOF-NIP powder was mixed with 5 mL of STH solution at a fixed initial concentration of 6.5 µM, with the mixture agitating by orbital shaking. Aliquots were removed at specified time intervals (5 to 60 min). For the isotherm experiments, the same ratio of solid to liquid was maintained, while the initial STH concentration varied from 5 µM to 40 µM. Samples were allowed to reach equilibrium over a fixed contact time of 30 min, as determined from the kinetics studies. In both cases, the pH of the solution was adjusted to 7.0. After the pre-determined time of adsorption, the mixture was separated by centrifugation at 10,000 rpm for 5 min, and the amount of STH in the solution supernatant was determined by a HPLC method using the relevant procedure [46]. The equilibrium adsorption capacity, *Q*_e_ (mg·g^−1^), is calculated from the equation Qe = $\frac{C_{0}-C_{e}}{mV}$, in which *C*_o_ (mg·L^−1^) and *C_e_* (mg·L^−1^) are the initial STH and residual STH concentrations in the solution, *m* (g) is the adsorbent mass, and V (L) is the volume of the STH solution.

### 2.6. The Experimental Procedures for Real Sample Analysis

Wastewater samples were collected from the local municipal wastewater treatment plant and filtered by a 0.45 μm membrane to remove suspended solids. The filtered samples were stored at 4 °C and analyzed within 24 h; prior to analysis, 1 mL of the filtered wastewater sample was diluted to a final volume of 10 mL with phosphate buffer. Human blood serum samples were collected at Zonguldak Bülent Ecevit University Hospital and stored at −20 °C until analysis. Deproteinization was performed by adding 0.5 mL of serum to 1.0 mL of acetonitrile, followed by vortexing for 2 min and centrifugation at 5000 rpm for 30 min. The supernatant was diluted to 5 mL with phosphate buffer, pH optimized, and filtered through a 0.22 μm membrane prior to analysis. For evaluation of accuracy, both the wastewater and serum samples were spiked with known concentrations of STH at three levels. The measuring results of fluorescence sensing were also supported by the measurements with HPLC. Chromatographic separations were carried out on a C18 reversed-phase column using a mobile phase consisting of water–methanol (9:1, *v*/*v*) with 0.1% formic acid under isocratic conditions at a constant flow rate of 1 mL min^−1^. For this approach, sulfathiazole was detected by a UV detector at a 285 nm wavelength.

## 3. Results and Discussion

**Figure 1:**
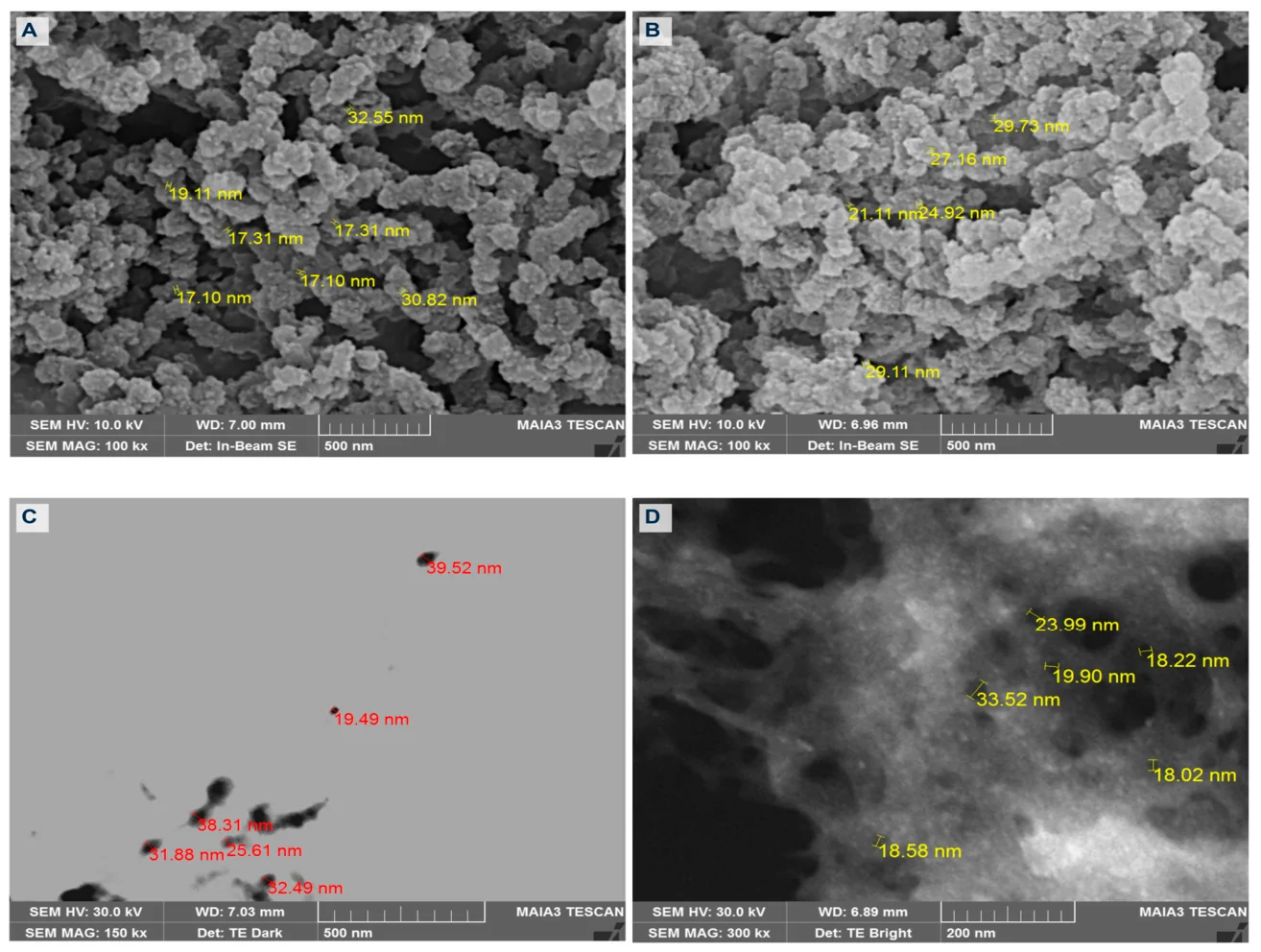
SEM images Mn-ZnS-MOF-MIP (**A**), Mn-ZnS-MOF-NIP (**B**), STEM images of Mn-ZnS (**C**), and Mn-ZnS-MOF-MIP (**D**).

As displayed in Figure 1A–D, the SEM and STEM clearly show that the Mn–ZnS–based materials were fabricated step by step. PEG-modified Mn–ZnS quantum dots consist of discrete, quasi-spherical nanoparticles, with the size ranging from 14.9 to 79.2 nm (mean diameter of 33.3 ± 13.0 nm), reflecting effective synthesis of quantum dots, as clearly demonstrated by the STEM image of Mn-ZnS in Figure 1C and Figure S1. Upon incorporation into the MOF matrix, the Mn–ZnS–MOF clearly shows a rough, aggregated granular morphology as a typical characteristic of the MOF–QD hybrid structures in Figure S2. As illustrated in Figure 1A, after molecular imprinting and template removal, the Mn–ZnS–MOF–MIP clearly displays a marked increase in porosity and fragmentation of the surface, with reduced particle sizes and visible interparticle voids, thus providing direct morphological evidence for the generation of imprinting-induced cavities while the structural integrity of the MOF framework is preserved. In sharp contrast, Mn–ZnS–MOF–NIP presents a much denser and compact morphology, with fewer open pores, in Figure 1B, thus emphasizing the role of a template in generating accessible recognition sites. The porous, sponge-like network observed in the imprinted composites, along with uniform dispersion of Mn–ZnS nanoparticles in Figure 1D, thus fully complied with successful incorporation of quantum dots, MOF formation, and effective molecular imprinting.

**Figure 2:**
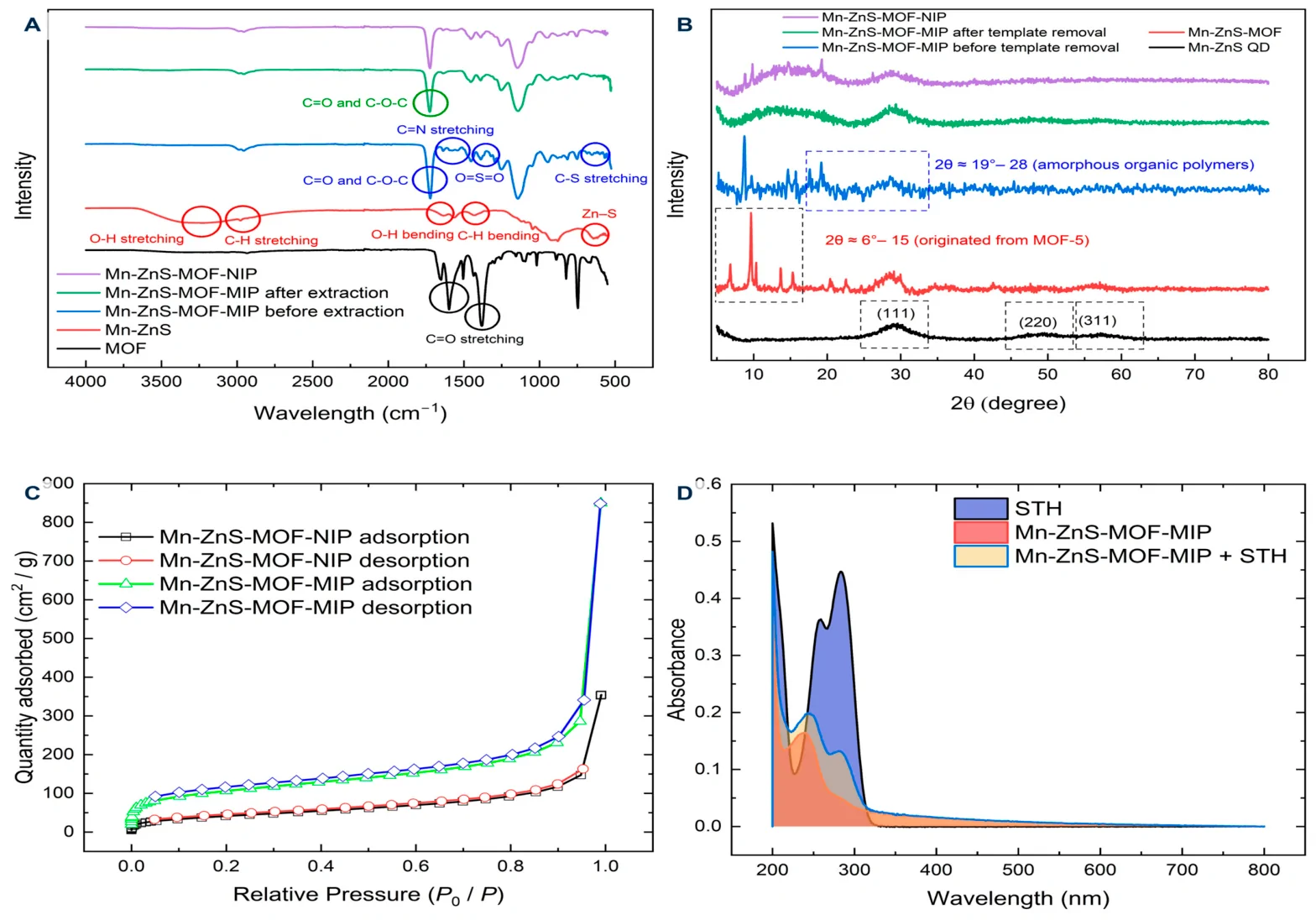
FTIR spectra (**A**) and X-ray diffraction patterns (**B**) of MOF, Mn-ZnS, Mn-ZnS-MOF, Mn-ZnS-MOF-MIP before and after template removal, and Mn-ZnS-MOF-NIP. Nitrogen adsorption and desorption curves (**C**) of Mn-ZnS-MOF-MIP and Mn-ZnS-MOF-NIP. UV spectra of STH, Mn-ZnS-MOF-MIP, and Mn-ZnS-MOF-MIP + STH (**D**).

As displayed in Figure 2A, FTIR analysis was also performed to verify every single step of the fabrication of nanocomposite sensing platform. In the FT-IR spectrum of MOF, the strong bands seen for the asymmetric–symmetric stretching vibrations of the carboxylate groups of C=O in the structure of the organic compound terephthalic acid coordinated with Zn(II) ions occur at wavenumbers of 1597 cm^−1^ and 1380 cm^−1^ [47]. In the FT-IR spectrum of Mn-ZnS, the wide absorption band at around 3229 cm^−1^ may be correlated with the O–H stretching vibrations stemming from hydroxyl groups in PEG chains. The absorption observed at 2970 cm^−1^ may be correlated with C–H stretching vibrations in CH_2_ group structures [42]. Another absorption at around 1634 cm^−1^ may correspond to O–H bending vibrations, while the absorption at around 1434 cm^−1^ may be correlated with C–H bending vibrations, and also the characteristic absorption at around 636 cm^−1^ may be correlated with Zn–S vibrational modes [48,49]. The prominent absorption bands in the IR spectra obtained for the Mn–ZnS–MOF-MIP (and the respective NIP) occurred at around 1722 (1725) cm^−1^ and at around 1143 (1144) cm^−1^ may be correlated with C=O and C–O–C groups originating from ethylene glycol dimethacrylate (EGDMA); in other words, it may be correlated with EGDMA residues incorporated in the material as a crosslinking agent in the course of polymerization processes [42,50]. Significantly, the band present at 1543 cm^−1^ (1528 cm^−1^ for free STH in Figure S3), which is characteristic of the MIP, is due to C=N stretching of the heterocyclic ring of sulfathiazole, which is not present in the spectrum of NIP. The weak absorbance present at 1319 cm^−1^ (1322 cm^−1^ for free STH), which is characteristic of MIP, is attributed to the asymmetric O=S=O stretching, whereas the weak absorbance present at 683 cm^−1^ (677 cm^−1^ for free STH), characteristic of MIP, is attributed to the C–S stretching of the sulfonyl group of sulfathiazole [51], which is again absent in the spectrum of NIP and even in that of the extracted MIP, confirming that the template molecule, STH, is duly eliminated from its matrix. The shifts in free STH peaks, compared to the sensor molecule, are due to intermolecular interactions of STH.

Figure 2B displays the XRD patterns of Mn-ZnS QDs, Mn-ZnS-MOF composite nanostructures, Mn-ZnS-MOF-MIPs prepared via both approaches, template-free scheme with eventual removal of the templates (MIPs), and their respective non-imprinted system (Mn-ZnS-MOF-NIP). The XRD pattern of Mn(II) ions doped ZnS QDs displays broad diffraction peaks around 2θ = 29°, 49°, and 57°, relating to (111), (220), and (311) lattice planes of cubic zincblende structure for ZnS. There was no evidence of secondary phase including MnS or manganese oxide molecules, suggesting successful doping of Mn-II ions into ZnS lattice, with no phase separation [52,53]. Overall, these XRD patterns convincingly reveal successful preparation of Mn(II) ion-doped ZnS QDs with retention of nanocrystallinity. On close inspection of the diffraction pattern of Mn-ZnS-MOF composite nanostructures, a merged pattern was obtained. There was retention of broad diffraction peaks with reduced scattering density for Mn-ZnS QDs. There were additional diffraction peaks located in the low-angle region (2θ ≈ 6–15°) that are indicative of a typical highly crystalline structure comprising prototype MOF; these positions correspond closely to characteristic reflections confirmed independently for pristine MOF-5 [54,55,56] (Figure S10). However, the coexistence of both types of peaks, without significant peak shifting, corresponding to the MOF, indicates the successful preparation of the composite, where the Mn-ZnS QDs are likely embedded within MOF without compromising its integral form [57,58]. Furthermore, the presence of such peaks in the composite with Mn-ZnS indicates that the MOF structure remains intact, but the reduced intensity of the peaks suggests the partial occupation of pores by the embedded QDs. Before the template removal, the XRD patterns of the composite following the polymerization process are highly changed. There are reduced sharp diffraction peaks for the MOF, suggesting the degradation or amorphic structure of the crystalline MOF phase via the polymerization process, or its chemical surroundings, where the diffraction patterns are highly reduced [59]. In addition, there are prominent humps centered at 19° and 28°, related to the unique features of amorphous organic polymers, together with the fingerprints of Mn-ZnS QDs, that are covered by this broad amorphous hump. Patterns for the MIP after the template extraction and the non-imprinted polymer (NIP), as expected, are quite similar to the pattern before removal. In both patterns, the broad amorphous peak has appeared without the restoration of MOF. This proves that the MOF structure is unstable under the conditions of the polymerization and has turned irreversibly to the amorphous composite material.

The nitrogen adsorption and desorption isotherms at 77 K were used to determine and analyze the surface area and porosity of imprinted and non-imprinted polymeric materials, which are shown in Figure 2C. The surface area of Mn-ZnS-MOF-MIP and Mn-ZnS-MOF-NIP, using Brunauer–Emmett–Teller, was revealed to be 380.13 m^2^ g^−1^ and 152.10 m^2^ g^−1^, respectively. This shows that MIP has a much higher surface area, thus confirming and proving that the procedure of molecular imprinting was effective and successful in creating specific recognition sites in the polymeric structure, which was formed due to the extraction of template molecules. The lack of a certain structure in Mn-ZnS-MOF-NIP, therefore, led to a much lower surface area.

It is well known that the predominant fraction of sensor platforms is driven by the principles of fluorescence quenching, primarily through dynamic or static mechanisms. In the process of static quenching, a fluorescent and a target molecule can form a complex in the ground state, thereby preventing the emission of light, which leads to significant changes in the UV spectrum [60]. As illustrated in Figure 2D, the combination of the UV–Vis and fluorescence data suggests that quenching of STH binding to the Mn–ZnS–MOF–MIP predominantly occurs by static quenching, resulting from complexation in the ground state inside the imprinted cavities [60,61,62]. Upon rebinding of STH, an UV–Vis spectrum that displayed distinct band broadening and intensity enhancement in the 230–300 nm region showed that the electronic transitions of STH were disturbed due to its incorporation into the polymeric environment rather than merely freely coexisting in solution. Such spectral changes correspond to a specific host–guest association and prove the formation of a nonfluorescent complex between STH and MIP. This behavior is further underlined by the strong and reproducible suppression of Mn–ZnS emission intensity following STH rebinding, which is proportional to the degree of occupancy of the imprinted sites and incompatible with a purely dynamic, collision-controlled process. Since STH shows negligible absorbance in the Mn–ZnS QDs emission region, Förster energy transfer can be excluded [63,64]. Quenching is then most plausibly driven by static complexation, with accompanying photoinduced electron transfer (PET) or localized electronic coupling within the binding cavities. The combination of all data confirms that Mn–ZnS–MOF–MIP operates by a static, binding-mediated quenching pathway, in accordance with the selective and cavity-dependent sensing behavior of the imprinted material.

**Figure 3:**
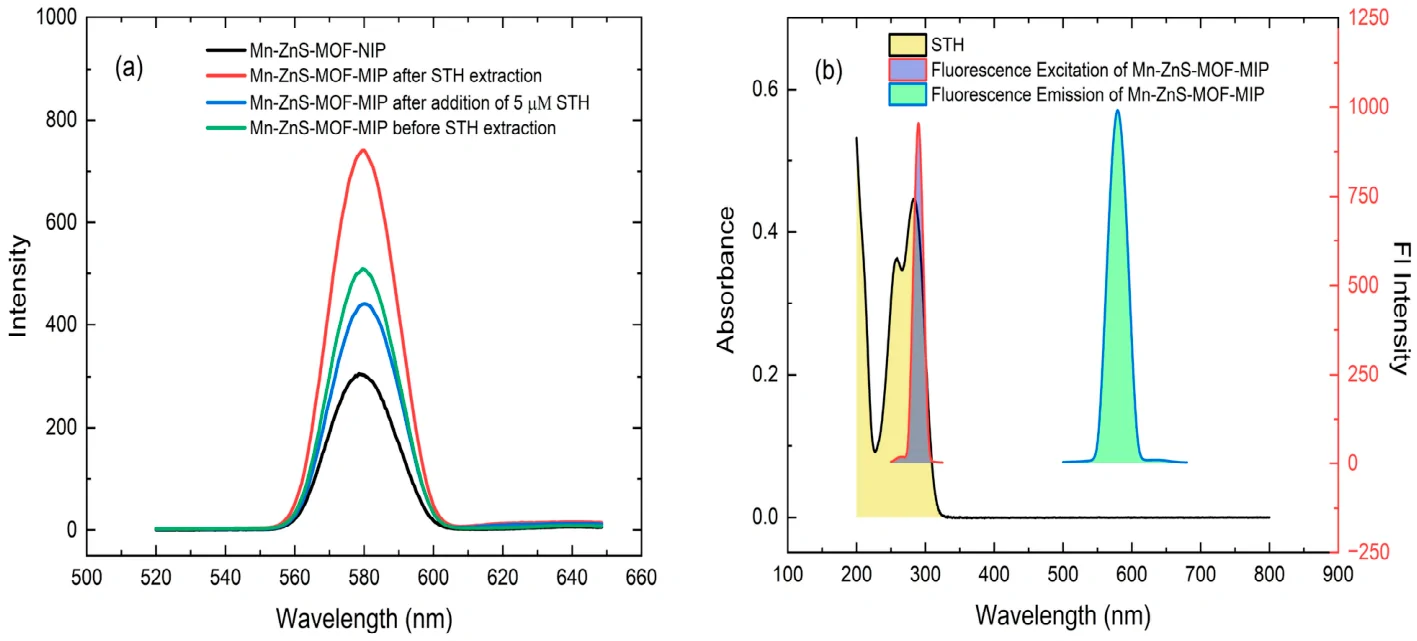
Fluorescence spectra of Mn-ZnS-MOF-NIP, Mn-ZnS-MOF-MIP before and after removal of template, and template-free Mn-ZnS-MOF-MIP after addition of 5 μM of STH (**a**). UV–vis absorption spectra of STH, and fluorescence excitation and emission spectra of Mn-ZnS-MOF-MIP (**b**).

These fluorescence emission spectra given in Figure 3a represent the synthesis, template removal, and rebinding performance of the analyte to the developed Mn-ZnS-MOF-MIP sensor for STH. Mn-ZnS-MOF-MIP, prior to STH removal, exhibits a quenched fluorescence signal, confirming that the STH template was successfully incorporated into the Mn-ZnS QDs during the imprinting process, suppressing their intrinsic luminescence. After template removal, substantial enhancement in fluorescence intensity was obtained. Such a “fluorescence turn-on” effect signals the creation of accessible, STH-complementary binding cavities within the polymer matrix. The subsequent rebinding experiment confirms the specificity of these imprinted sites. As clearly shown, after the subsequent addition of 5 µM STH to the extracted MIP, significant fluorescence quenching behavior was observed. This “turn-off” response was thus a result of the selective reoccupation of the cavities by STH molecules, enabling an efficient electron or energy transfer process from the excited Mn-ZnS QDs to the analyte. For comparison, the fluorescence intensity of the non-imprinted polymer (NIP) is comparable to that of the MIP before template extraction. This equivalence confirms that the polymerization process itself does not inherently quench the QD fluorescence. The substantially lower quenching behavior of NIP upon exposure to STH provides the validation that the fluorescence “turn-off” response in the MIP is not a feature of nonspecific adsorption but is directly related to specific molecular-recognition events enabled by the imprinting process. The stability of the characteristic Mn^2+^ emission maximum (~590 nm) across all spectra confirms that the QD’s optical properties are preserved within the composite architecture. These results together confirm the successful construction of a selective sensor. Such reversible “on–off” fluorescence switching between extracted and analyte-bound states of MIP provides a clear and reliable signal transduction mechanism for the quantitative detection of STH.

To isolate the contribution of each individual component to the sensing response, the fluorescence quenching efficiencies of bare Mn-ZnS QDs, Mn-ZnS-MOF, Mn-ZnS-MIP (synthesized without the MOF layer), and the complete Mn-ZnS-MOF-MIP composite were compared at a fixed STH concentration (1.66 µM) (Figure S11). Bare Mn-ZnS QDs showed minimal quenching (3.6%), consistent with weak, nonspecific interaction with STH. The porous MOF host alone raised this to 8.8%, and the imprinted MIP layer alone gave a larger response of 11.8%, reflecting the contribution of selective molecular recognition. The complete Mn-ZnS-MOF-MIP composite gave the highest response of all (15.1%), a 28% increase over the MOF-free Mn-ZnS-MIP material. This shows that the MOF adds a measurable benefit beyond what the MIP recognition layer provides alone, consistent with its proposed role in pre-concentrating STH near the QD surface and increasing local analyte availability at the imprinted binding sites.

The photophysical mechanism responsible for the selective fluorescence quenching of the Mn-ZnS-MOF-MIP sensor by sulfathiazole (STH) was probed by correlating the absorption profile of the analyte with the excitation and emission characteristics of the sensor material. The combined optical data indicate that STH binding to the Mn–ZnS–MOF–MIP produces fluorescence quenching through a binding-mediated (static) mechanism with a likely secondary contribution from an inner-filter effect (IFE) [65]. Two independent observations support this assignment. First, as shown in Figure 3b, the UV–Vis trace of STH shows strong absorption in the UV region (≈230–300 nm), which coincides with the measured excitation profile of the Mn–ZnS–MOF–MIP. This spectral overlap means that free or bound STH can attenuate the excitation reaching the Mn–ZnS emitters and thus produce apparent quenching via IFE when present in solution or bound at the MIP interface [60,62]. Second, the UV spectrum of STH associated with the MIP displays broadening of the STH bands. Similarly, the previously mentioned reversible, imprint-dependent changes in steady-state emission suggest the formation of a ground-state host–guest complex within the imprinted cavities. The ground-state association reduces the fraction of fluorescent or uncomplexed emitters and is characteristic of static quenching rather than dynamic quenching [66]. Mechanistically, static quenching in this system may arise from strong local interactions—hydrogen bonding, π–π or electrostatic contacts, and electronic environment of the Mn–ZnS centers; within the bound complex, nonradiative deactivation pathways—including short-range photoinduced electron transfer (PET)—are plausible and could further suppress emission [67,68]. Since Förster resonance energy transfer is not supported by the spectra—there is no absorption by STH in the Mn-ZnS emission region—FRET is unlikely to contribute. Collectively, data are most consistent with a primary static quenching channel—that is, ground-state complex formation—supplemented by IFE stemming from excitation-band overlap; PET or other short-range electron/hole transfer processes cannot be excluded but require further corroboration. MOF-driven preconcentration of STH within the imprinted cavities amplifies the magnitude of the fluorescence response by increasing the effective local analyte concentration at the QD surface, while the direction of that response is governed by the specific photophysical relationship between STH and the Mn–ZnS QD. Because STH shows negligible absorbance in the QD emission region and does not itself re-emit following interaction with the excited QD, its predominant effect is to provide a non-radiative relaxation pathway via static complexation, manifesting as quenching rather than turn-on enhancement.

Further mechanistic evidence came from Stern–Volmer analyses performed at 35 and 45 °C (Figures S12 and S13). As displayed in Figure S14, the quenching constant (*K*_sv_) fell steadily as temperature rose, a pattern more consistent with static than dynamic quenching. Because higher temperatures destabilize the ground-state STH–sensor complex, the binding equilibrium is pushed toward dissociation, so less quenching occurs at a given STH concentration. Considered alongside the evidence presented earlier, this temperature dependence points directly to a static, binding-mediated quenching mechanism.

Given the architecture underlying this sensor design, in which a thin, conformal MIP shell is grown directly around the porous MOF-encapsulated Mn–ZnS QDs via surface imprinting, the STH-selective cavities generated during synthesis are not physically isolated from the QD surface. Because the MIP layer is kept thin and surface-accessible—in contrast to the deeply buried, mass transfer-limited sites typical of bulk-polymerized MIPs—and because the underlying MOF is itself porous and designed to concentrate STH within its nanopores, the imprinted binding cavities are functionally continuous, with the MOF pore network leading to the QD surface. STH captured within an imprinted cavity is therefore positioned at, or in direct communication with, the MOF–QD interface rather than remaining isolated at the outer polymer surface. This spatial arrangement is consistent with the proposed static, binding-mediated quenching mechanism, in which STH forms a ground-state complex in sufficiently close proximity to the Mn–ZnS emitters to enable short-range electronic coupling and photoinduced electron transfer, alongside a secondary inner-filter-effect contribution that does not require direct contact with the QD surface.

### 3.1. Optimization of Sensing Conditions

**Figure 4:**
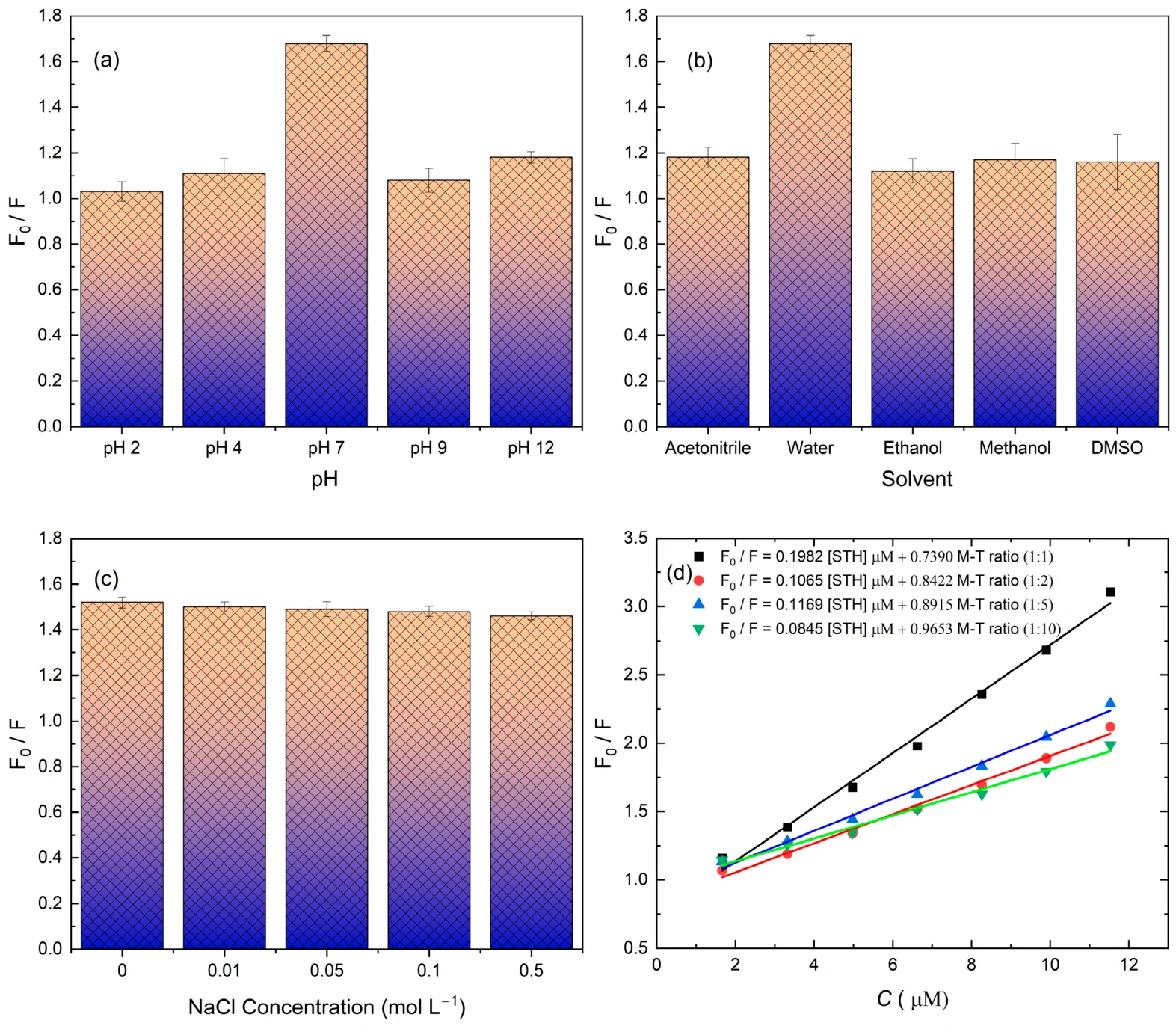
Fluorescence intensity of the Mn-ZnS-MOF-MIP recorded for the investigation of the effect of pH (**a**), effect of solvent (**b**), and effect of ionic strength (**c**) in the presence of 5 µM STH, and the effect of monomer–template ratio (**d**).

In an attempt to reach the maximum sensitivity and stability, the operational environment of the developed sensor was systematically optimized by examining the critical parameters of solvent polarity, pH, ionic strength, and monomer/template ratio, and the obtained findings are demonstrated in Figure 4. Among them, pH is regarded as the factor of leading concern since it can affect both the charge state of STH and the binding process of the sensing device to STH. The sensor response showed a trend of systematic and strong pH dependence. As clearly seen in Figure 4a, the signal increased gradually from acidic pH values and reached a maximum at pH 7; further increase in pH higher than 9 resulted in a decline under alkaline conditions. Such a profile is indicative of the fact that the sensing mechanism is driven by protonation–deprotonation equilibria of the sensor and/or analyte. At low pH, the protonation of active sites might restrain the electron transfer, or the number of hydrogen bonds established between the analyte located on the sensing platform and the template seems to decrease, which in turn collectively reduces the measurable response. Deprotonation at pH higher than 7 most probably destabilizes the recognition interaction or creates minor surface defects, which overall result in decreasing sensitivity. It could also be quite noticeable that the performance of the sensor is considerably dependent on the solvent environment. As shown in Figure 4b, of all the solvents tested, water yielded the highest response, with the signal amplifying well above those recorded in acetonitrile, ethanol, methanol, and DMSO. An increase in performance in water is related to more effective solvation both of the analyte and the sensor active sites, enabling more efficient charge transfer and the stabilization of the complex. All of these results point unequivocally to water as an optimal solvent to achieve the maximum sensor sensitivity. The response of the sensor was further investigated under different ionic-strength conditions using NaCl concentrations in the range of 0–0.5 mol L^−1^ (Figure 4c). The response of the sensor was further investigated under different ionic-strength conditions using NaCl concentration in the range of 0–0.5 mol L^−1^. Contrary to the case of solvent and pH effects, no significant signal-intensity changes were recorded in the whole ionic-strength range, as proven in Figure 4c. Indeed, the sensor sustained a comparable output regardless of the salt concentration, which accordingly means that the recognition process and electron transfer occurring are almost insensitive to electrostatic screening in the tested conditions. This stability suggests that the sensor’s interaction mechanism is not strongly dependent on long-range electrostatic forces or that any ionic screening effects are effectively compensated by the structural or electronic properties of the sensing interface. Such signal robustness against changes in ionic environment is beneficial for practical applications in that moderate variation in sample salinity would not compromise analytical performance.

To rationalize how monomer-to-template stoichiometry influences sensor performance, MIPs were prepared using 1:1, 1:2, 1:5, and 1:10 monomer-to-template ratios. Fluorescence-based calibration curves for all of these formulations were recorded over the concentration range of 1.6–12 μM STH, together with corresponding NIP responses. Sensitivities, obtained from the slopes of the calibration graphs (Figure 4d), and IFs calculated as the ratio of MIP:NIP sensitivities were used to obtain quantitative estimations of the analytical efficacy and imprinting efficiency. The imprinting factor, *IF*, was defined as *IF* = *S*_MIP_/*S*_NIP_, where *S* is the slope of the linear calibration curve. A higher *IF* reflects higher specific binding capacity of the MIP compared to nonspecific adsorption by the NIP. The computed *IF* values showed a strong dependence on the synthesis ratio: ratio 1:1, *if* = 4.8; ratio 1:2, *if* = 2.5; ratio 1:5, *if* = 2.8; and ratio 1:10, IF = 2.05. The results obtained show a strong imprinting efficiency dependence on monomer/template stoichiometry. Indeed, the 1:1 ratio provided the highest *IF* value, *IF* = 4.8, which indicates the strongest enhancement of selective binding with respect to the NIP. This could be interpreted as evidence that equimolar monomer/template conditions favor the formation of well-defined and highly specific binding cavities. Under this ratio, the template is likely fully and effectively surrounded by functional monomers during polymerization, allowing for the maximum number and orientation of the interaction points, while yielding the most structurally accurate imprinted sites. Increasing this relative amount of template beyond the equimolar point (ratios of 1:2 and 1:5) gave substantially reduced *IF* values, *IF* = 2.5 and 2.8, respectively. The diminished imprinting efficiencies are due to overcrowding of the template molecules, so that the regular arrangement of monomers in the pre-polymerization mixture is disrupted, with incomplete or poorly formed binding cavities. Under excess template conditions, polymerization may proceed without each template molecule being uniformly enveloped by monomers. This yields a generation of binding sites that are heterogeneous with regard to size, geometry, and affinity. The *IF* was further decreased at the most diluted monomer condition, *IF* = 2.05 for a 1:10 ratio, confirming the inadequacy of monomer availability for effective cavity formation. Under these conditions, the relative scarcity of functional monomer with respect to template results in inadequate stabilization of template–monomer complexes, giving a polymer matrix that contains a large number of non-functional or poorly defined cavities. Consequently, such polymers exhibit poorer selectivity and performance, approaching that of the NIP. These experiments point out clearly that the 1:1 stoichiometry of the monomer/template represents, in fact, the optimal formulation when considering the quality of the imprinted sites and the strength of the selective recognition behavior. This stoichiometry was, therefore, adopted for all subsequent steps related to sensor fabrication.

### 3.2. Adsorption and Kinetics Studies

**Figure 5:**
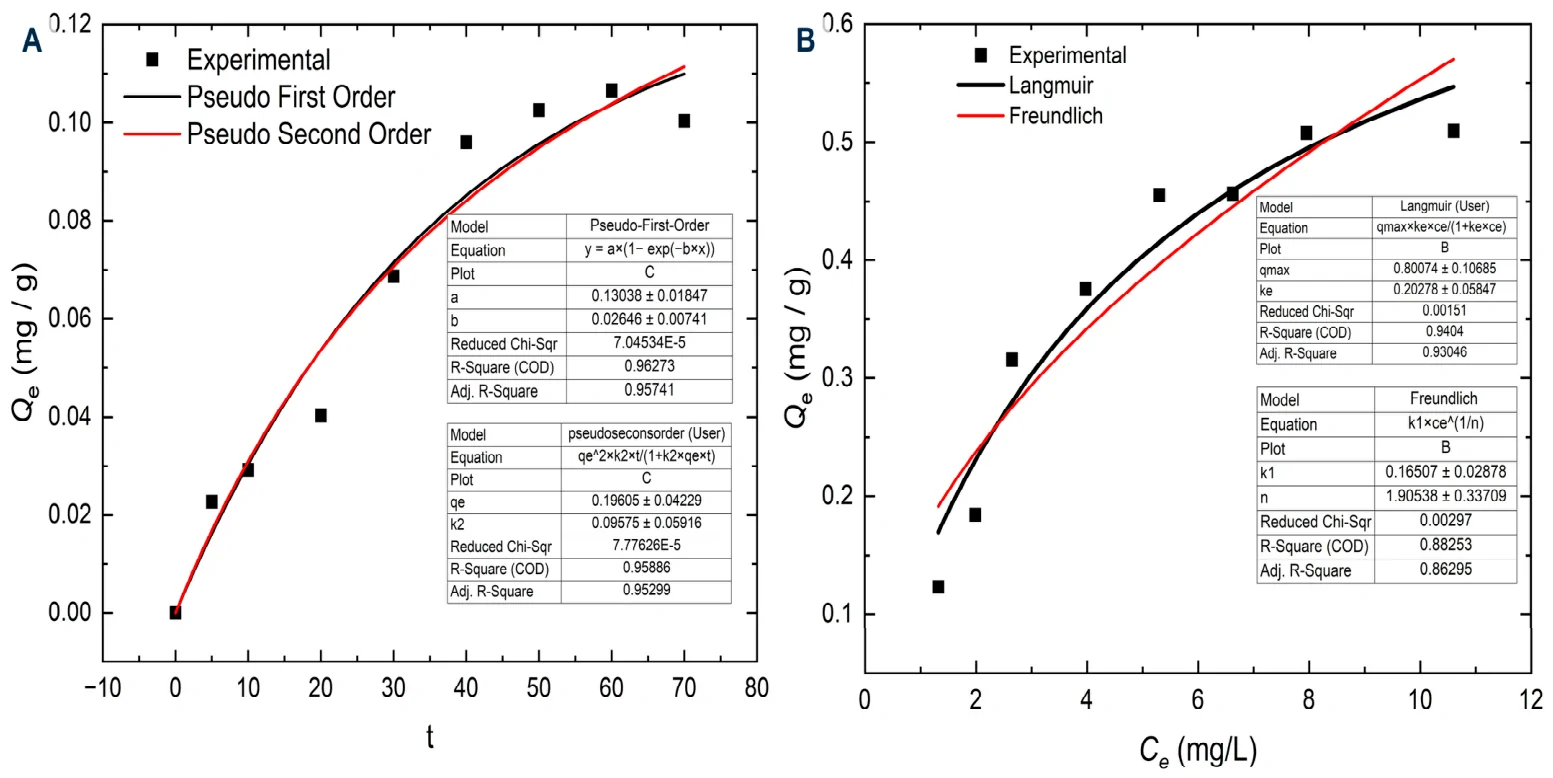
The experimental kinetics for the STH adsorption on Mn-ZnS-MOF-MIP and experimental data fitted to the pseudo-first-order and pseudo-second-order model (**A**). Experimental and fitted Langmuir and Freundlich isotherms for STH adsorption onto Mn-ZnS-MOF-MIP (**B**).

The kinetic process of sulfathiazole (STH) on the Mn-ZnS-MOF-MIP surface was examined employing pseudo-first-order (PFO) and pseudo-second-order (PSO) kinetics. The graph of the experimental data and nonlinear fit of PFO and PSO kinetics are shown in Figure 5A.

Pseudo-first-order model:

(1) $$Q_{t}=Q(1-e^{-K_{1}t})$$

Pseudo-second-order model:

(2) $$Q_{t}=\frac{Q_{e}^{2}K_{2}t}{1+Q_{e}K_{2}t}$$

where the adsorption capacity at time *t* and at equilibrium is represented by *Q*_t_ and *Q*_e_ (mg g^−1^), and the corresponding rate constants are *K*_1_ (min^−1^) and *K*_2_ (g mg^−1^ min^−1^).

From the kinetic graphs, it can be seen that both models are able to reflect the overall STH uptake tendency correctly. On careful analysis of the statistical parameters, it is found that the pseudo-first-order model is slightly better than the other in terms of data fit. The correlation coefficient (R^2^) of the PFO model (R^2^ = 0.95097) is higher than that of PSO (R^2^ = 0.93696), and the reduced chi-square value of the PFO model (chi-square = 0.00214) is lower than that of PSO (chi-square = 0.00275). Hence, it can be concluded that the experimental data of STH adsorption are more correctly described by the pseudo-first-order kinetic mechanism. Also, the equilibrium STH adsorption capacity predicted by the pseudo-first-order kinetic mechanism (*Q*_e_ = 0.5168 ± 0.0527 mg/g) matches more closely to the experimental STH adsorption plateau (approx. 0.45–0.48 mg g^−1^) than the value predicted by PSO (*Q*_e_ = 0.7312 ± 0.1410 mg g^−1^), which significantly exceeds the experimental plateau.

Further, the difference in correlation coefficients between the two models further validates the suitability of the PFO model to describe the STH adsorption process on the Mn–ZnS–MOF–MIP surface. The better applicability of the PFO model suggests that the total process of adsorption is controlled by the concentration difference between the liquid phase and the solid surface, which is particularly relevant in diffusion-governed processes, like physical adsorption [69]. The PSO model, which is generally known to be representative of chemisorption processes, which are responsible for bonding between the surfaces [70], has less correlation, signifying the absence of such chemical reactions at the surface influencing the rates of the overall process of adsorption. This further correlates to the efficient utilization of the designed composite, wherein the porous MOF matrix facilitates diffusion, thereby providing a selective binding possibility using the molecule-imprinted part of the matrix. Nevertheless, the fact that the PSO model fits fairly well suggests that specific interactions related to the molecular-recognition sites continue to play a role in the adsorption process, although they are no longer the rate-determining step. The overall adsorption kinetic behavior can therefore be attributed to a combined adsorption mechanism, wherein diffusion-controlled physisorption is more predominant, and specific interactions are more related to enhancement of selectivity rather than affecting the rate of adsorption [69].

The equilibrium adsorption process of sulfathiazole (STH) on Mn–ZnS–MOF–MIP was further studied using the Langmuir isotherm equation and the Freundlich isotherm equation in order to understand the character of the adsorption surface, as well as the interaction mechanism between STH molecules and the imprinted material.

Langmuir and Freundlich isotherm equations are mathematically described, respectively, as follows:

(3) $$Q_{e}=Q_{max}\frac{K_{e}C_{e}}{1+K_{e}C_{e}}$$

(4) $$Q_{e}=k_{f}C_{e}^{1/nF}$$

where *Q*_e_ is the equilibrium adsorption capacity (mg g^−1^), *C*_e_ is the equilibrium concentration of STH in the solution (mg L^−1^), *Q*_max_ is the maximum theoretical monolayer adsorption capacity (mg g^−1^); *K*_e_ is the Langmuir equilibrium constant; and *k_f_* and *n_F_* represent the Freundlich adsorption constant and the heterogeneity factor, respectively.

The experimental result and the nonlinear fitting result are plotted in Figure 5B. As can be seen from the figure, the Langmuir isotherm equation is in better agreement with the experimental result compared to the Freundlich isotherm equation. This can be seen from the higher values of the correlation coefficient for the Langmuir isotherm equation (R^2^ = 0.9404) rather than the Freundlich isotherm equation (R^2^ = 0.8825), as well as the lower values of the reduced chi-square (0.00151 rather than 0.00297). The very good conformity with the Langmuir isotherm (R^2^ > 0.93) indicates that STH adsorption predominantly occurs through monolayer adsorption on a homogeneous surface with equivalent binding sites. This is one of the features of molecularly imprinted polymers, in which the template molecule (STH) provides identical binding sites through the formation of cavities during polymer synthesis. The calculated “separation factor” (*R*_L_ = 1/(1 + K_L_C_0_)) for studied concentrations varies from 0.33 to 0.83, verifying the good adsorption conditions (0 < R_L_ < 1) [71,72]. The Freundlich equation, which considers multi-layer adsorption on a heterogeneous surface [73], is a comparatively poorer fit. The Freundlich parameter *n* = 1.91 (1/*n* ≈ 0.52) indicates the medium intensity of adsorption, but the lower correlation factor points to the fact that heterogeneity of the surface is not the predominant feature defining STH adsorption in this particular system. Even if the adsorption capacity of 0.80 mg/g is moderate, the major role of the MOF molecularly imprinted polymers is not the uptake capacity, but rather selective molecule recognition. As revealed by previous research, the presence of MOFs primarily provides a large surface area for the support as a means of facilitating mass transport resistance, while the selectivity of the analyte is given by the presence of the surface-imprinted polymer layer [74]. The creation of binding sites within the outermost layer of the polymers explains the prevailing Langmuir-type isotherm behavior observed for the current system, in spite of the inherent heterogeneity of the MOF surface.

Adsorption capacity and isotherm were also examined and compared to NIP given in the Supplementary Information, as illustrated in Figures S15 and S16. The adsorption capacity of MIP is higher than that of NIP, which implies effective binding sites and cavities were created for MIP. In Figure S15, the adsorption capacities of MIP and NIP sharply rose within the first 30 min and then plateaued at the same level. A similar trend but higher level of change represents higher mass transfer rate and stronger binding ability for MIP [43].

### 3.3. Sensitivity, Selectivity, Reproducibility, and Stability

**Figure 6:**
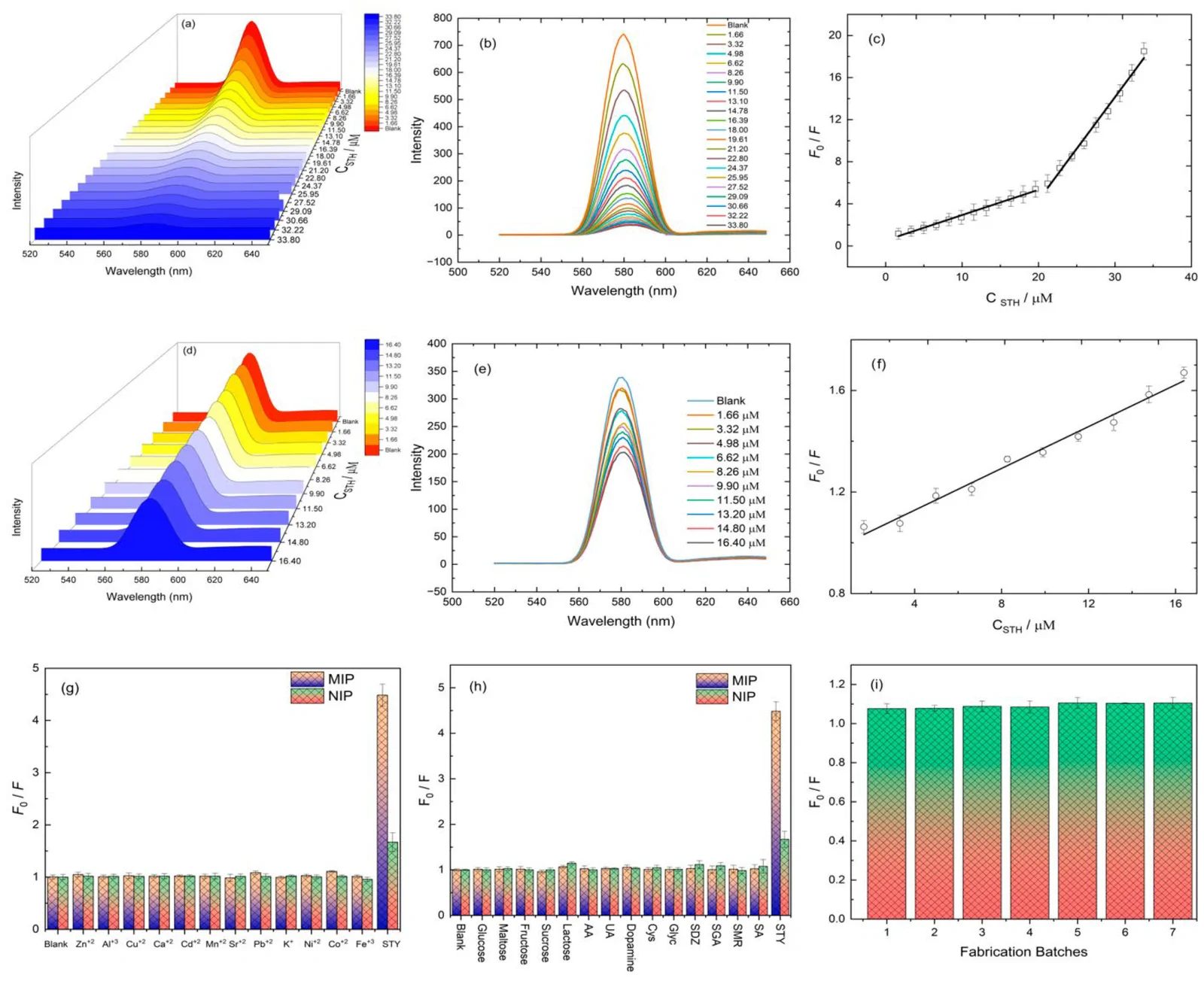
Color stereo fluorescence data and raw fluorescence data after addition of STH to the Mn-ZnS-MOF-MIP (**a**,**b**) and Mn-ZnS-MOF-NIP (**d**,**e**). Linear curves of the *F*_0_/*F* of the Mn-ZnS-MOF-MIP (**c**) and Mn-ZnS-MOF-NIP (**f**). Fluorescence responses of Mn-ZnS-MOF-MIP and Mn-ZnS-MOF-NIP in the existence of STH or interfering metal ions and analytes (**g**,**h**). The reproducibility of Mn-ZnS-MOF-MIP fluorescent probes for the determination of STH (**i**).

The analytical performance and binding properties of the sulfathiazole-imprinted (MIP) and non-imprinted (NIP) sensors were investigated quantitatively by fluorescence quenching experiments, based on the ratio of the initial to the measured fluorescence intensity (*FI*) signals. *FI* intensity of the (*F*_0_/*F*) was measured and graphed against an increasing concentration of STH (Figure 6a–c). A biphasic binding response was shown by the MIP sensor, typical of the presence of heterogeneous binding sites with unequal binding affinities to the target analyte. For the lower concentration range of the analyte (1.6 to 19.6 µM), the linear response was described by the equation *F*_0_/*F* = 0.2404*C* + 0.5263 (R^2^ = 0.9924). It defines the region of high-affinity binding of the STH molecules to the designed molecular cavities formed in the imprinting process. The limit of detection (LOD) was calculated using the equation LOD = 3s/k, where s is the standard deviation of five blank measurements, and k is the slope of the calibration curve, yielding an extremely low value of 13.75 nM. The high sensitivity value also confirms the successful creation of recognition sites by the imprinting process. At higher concentrations (22.2–33.8 µM), a second, steeper linear region was detected with the regression equation of (*F*_0_/*F* = 0.9799*C* − 15.26; R^2^ = 0.9916) This significant change in the slope represents the saturation of the high-affinity sites and the occupancy of the lower-affinity, nonspecific sites, typical of heterogeneous MIP surfaces. As shown in Table S1, the proposed Mn–ZnS/MOF/MIP sensor exhibits sensitivity comparable to or better than most fluorescence-, colorimetric-, and chromatography-based methods reported to date [16,75,76,77,78,79], as given in Table S1, while offering the added advantages of dual-selectivity recognition through the combined MOF and MIP layers, along with a simple, rapid, and visual fluorescence readout that avoids the more elaborate instrumentation required by other platforms. Although several reported sensors exhibit lower absolute LOD values, direct comparison is complicated by the fact that, to our knowledge, no three-component (QD/MOF/MIP) sensor analogous to ours has been described for sulfathiazole detection; the platforms listed instead represent single- or two-component alternatives. The synergistic integration of Mn–ZnS quantum dots, an MOF scaffold, and a molecularly imprinted polymer in this work confers distinct practical advantages beyond LOD alone: the sensor exhibits two coexisting linear response regions, enabling reliable quantification across a broad concentration range without requiring re-calibration or sample dilution; the imprinted cavities provide high molecular selectivity even in the presence of structurally related interferents. Several of the comparator methods rely on multi-step sample pretreatment prior to detection, whereas the present sensor achieves comparable or superior performance through a single fluorescence turn-off measurement, offering a simpler and faster workflow for routine analysis.

In contrast, the NIP sensor (Figure 6d–f), which has no specific recognition sites, showed linear response over the concentration range tested (1.6 to 16.4 µM), which can be expressed by *F*_0_/*F* = 0.0411*C* + 0.9638 (R^2^ = 0.9845). The slope value for this line is about 5.8-fold lower than that for the high-affinity part of the MIP. This provides direct evidence that the response of the MIP within its first linear range is based on specific molecular recognition. In addition, the value for the LOD of the NIP sensor was calculated to be 160 nM, which is more than one order of magnitude (∼11.6-fold) higher than that for the MIP. This emphasizes the high sensitivity achieved through the imprinting technique. In conclusion, the attained findings in unequivocally established the successful development of a selective MIP sensor for the analyte STH. The coexistence of the two linear slopes in the response of the MIP sensor ensures the presence of a nonuniform distribution of binding sites on the MIP matrix. The initial linear region of greater sensitivity (LOD of 13.75 nM) of the MIP sensor response is solely a result of the imprinted effect, while the NIP response highlights the prominent role of the imprinted sites in achieving the sensitivity and selectivity of the analyte.

While steady-state Stern–Volmer analysis alone cannot unambiguously distinguish static from dynamic quenching, the shape of the response profile offers additional mechanistic insight [80]. The *F*_0_/*F* response of the MIP resolves into two discrete linear regimes (Figure 6c) rather than a single smoothly curving profile. A continuously upward-curving Stern–Volmer plot is generally associated with a concentration-independent combination of static and dynamic quenching acting on a single fluorophore population [80]; the discrete breakpoint observed here is instead more consistent with sequential engagement of two distinct binding populations—high-affinity imprinted cavities approaching saturation near ~20 µM STH, followed by lower-affinity, nonspecific sites at higher concentrations. This picture is supported by the NIP control (Figure 6f), which retains the same Mn–ZnS emitters but lacks specific recognition cavities and shows a single, markedly shallower linear response; a substantial dynamic contribution would be expected to affect both materials similarly, so its comparative absence in the NIP response favors a predominantly static, site-specific mechanism in the MIP.

The molecular selectivity of the synthesized MIP toward STH was systematically investigated in comparison with structurally related sulfonamide antibiotics, common biological molecules, and relevant metal ions, which are 25 times more concentrated compared to STH, which can potentially interfere with the analytical response. From Figure 6g,h, it can be observed that the MIP presented a sharp and highly specific interaction with STH, which is reflected by the remarkable enhancement of fluorescence quenching ratio, *F*_0_/*F*. In contrast, all the remaining tested species—metal ions, including Zn^2+^, Al^3+^, Cu^2+^, Ca^2+^, Cd^2+^, Mn^2+^, Sr^2+^, Pb^2+^, K^+^, Ni^2+^, and Co^2+^; and molecules like glucose, fructose, sucrose, lactose, ascorbic acid, uric acid, glycine, cysteine, dopamine, SDZ, SGA, SMR, and SA—result in responses very close to that of the blank solution. Among all species tested, including STH, only STH gave a significantly higher variation from baseline with the MIP sensor, confirming that the imprinted cavities have high affinity and recognition capability for the target molecule. In contrast, NIP revealed only minor variation among all tested species, including STH, showing the absence of specific binding sites, thus confirming that the strong response observed with MIP originates from the imprinting process itself. The results clearly indicate, overall, excellent molecular selectivity of the MIP sensor toward sulfathiazole with rather negligible interference by structurally related sulfonamides, biological molecules, and metal ions. Such high discrimination ability confirmed the suitability of the developed MIP for reliable and selective determination of STH in complex sample matrices.

Being able to test the practicability of the developed STH–MIP sensor on a larger scale, a complete analysis of the reproducibility and stability of the device was also performed. Reproducibility of the fabrication procedure for the sensors was tested for seven different batches of MIP sensors made independently to a set concentration of STH. The results are represented in Figure 6i. The RSD value obtained for the experiment is 1.94%. A low RSD value indicates the robustness of the synthesis and sensor preparation methodology, which is essential for scalable research applications.

The long-term stability of a single MIP sensor toward a constant STH level, in terms of operational functionality, and regeneration performance over five cycles were also examined. The obtained results were also discussed and presented in Figures S17 and S18 in the Supplementary Information.

### 3.4. Real Sample Analysis

The applicability of the newly designed fluorescent sensor was tested using wastewater samples and serum samples, and the data were compared with that of a reference HPLC method, as presented in Table 1. All experiments were carried out in triplicate. The recoveries achieved in the wastewater samples differed from 90.00% to 101.04%, with the RSD levels falling below 2%, confirming the accuracy, as well as the high reproducibility, of the fluorescent sensing technique. The recoveries within the blood serum matrices ranged between 98.75% and 104.24%, with RSD below 1.66%, showing a very stable performance even with such a complex biological matrix. Comparison with the results of HPLC made it clear that the agreement between the values measured by the sensor and those measured by HPLC was good at all levels of concentration in the case of both matrices, confirming its usability for the fast and economical determination of STH, where routine chromatographic analysis may be impractical. 

## 4. Conclusions

In conclusion, a highly selective and sensitive fluorescent sensing platform was developed with the Mn–ZnS–MOF–MIP nanocomposite for detecting STH. For the first time, the deliberate triadic integration of Mn^2+^-doped ZnS quantum dots, a porous MOF scaffold, and a surface-imprinted polymer layer effectively overcame the intrinsic limitations of conventional QD-, MOF-, or MIP-based sensors when used separately. Thanks to the amplification of mass transfer and local analyte preconcentration by the MOF host, and the well-defined recognition cavities provided by the imprinted MIP layer, the proposed sensor enables fast and selective binding of STH and efficient signal transduction. The sensor exhibits a pronounced turn-off fluorescence response, with static quenching as the prevailing mechanism, a detection limit in the nanomolar range, high imprinting efficiency, and excellent discrimination against structurally related sulfonamides and common interferents. The sensor demonstrates good stability and reproducibility, in addition to its reusability, with reliable analytical performance in real wastewater and blood serum samples and with good agreement with HPLC analysis. Although the adsorption capacity is moderate, the results clearly point out that selective molecular recognition rather than maximum uptake controls the analytical performance of the system. This work provides an effective and universal sensing approach and outlines the general framework for designing high-performance fluorescence sensors for antibiotics and other emerging contaminants in complex environments.

## Figures and Tables

**Table 1:** The measured STH content in wastewater and blood serum matrices by designed fluorescent sensor and HPLC (n = 3).

		Fluorescent			HPLC		
Sample	Added (µM)	Found (µM)	Recovery (%)	RSD (%)	Found (µM)	Recovery (%)	RSD (%)
	1.60	1.54	96.25	1.84	1.67	104.38	1.20
**Wastewater**	3.30	2.97	90.00	1.87	3.27	99.09	0.92
	6.70	6.77	101.04	0.63	6.50	97.05	1.45
	1.60	1.58	98.75	0.90	1.65	103.12	1.25
**Blood Serum**	3.30	3.44	104.24	0.30	3.40	103.03	0.88
	6.70	6.80	101.49	1.66	6.40	95.52	0.78

## Data Availability

Raw Data supporting the conclusions of this article will be made available by the authors on request.

## Supplementary Materials

The following supporting information can be downloaded at https://www.mdpi.com/article/10.3390/bios16090459/s1, Figure S1: Size distribution histogram of Mn-ZnS quantum dots determined from STEM image analysis; Figure S2: SEM image of Mn-ZnS-MOF; Figure S3: The FTIR spectrum of STH; Figure S4: The FTIR spectrum of Mn-ZnS; Figure S5: The FTIR spectrum of MOF5; Figure S6: The FTIR spectrum of MOF-Mn-ZnS; Figure S7: The FTIR spectrum of Mn-ZnS-MOF5-MIP before removal of STH; Figure S8: The FTIR spectrum of Mn-ZnS-MOF5-NIP; Figure S9: The FTIR spectrum of Mn-ZnS-MOF5-MIP after removal of STH; Figure S10: XRD pattern of MOF5 [54,55,56]; Figure S11: Fluorescence emission spectra of (A) Mn-ZnS, (B) Mn-ZnS-MOF, (C) Mn-ZnS-MIP, and (D) Mn-ZnS-MOF-MIP before (black) and after (red) the addition of 1.66 µM STH; Figure S12: Fluorescence emission spectra of Mn-ZnS-MOF-MIP in the presence of increasing STH concentrations (0–11.5 µM) recorded at 35 °C; Figure S13: Fluorescence emission spectra of Mn-ZnS-MOF-MIP in the presence of increasing STH concentrations (0–11.5 µM) recorded at 45 °C; Figure S14: Temperature-dependent Stern–Volmer plots for Mn-ZnS-MOF-MIP at 25, 35, and 45 °C; Figure S15: The experimental kinetics for the STH adsorption on Mn-ZnS-MOF-NIP and experimental data fit-ted to the pseudo-first-order and pseudo-second-order model; Figure S16: Experimental and fitted Langmuir and Freundlich isotherms for STH adsorption onto Mn-ZnS-MOF-NIP; Figure S17: The long-term stability of the fluorescent probe. Figure S18: Reusability of the Mn-ZnS-MOF-5-MIP sensor for STH detection over repeated regeneration cycles. Figure S19: Fluorescence emission spectrum of the Mn-ZnS/MOF/MIP; Table S1: Comparison of the analytical performance of the Mn–ZnS/MOF/MIP fluorescent sensor with previously reported methods for the determination of sulfathiazole (STH) [16,75,76,77,78,79]; Chart S1: Chemical structures of the compounds evaluated in selectivity study corresponding to Figure 6h.
