# Supplementary material for: Triadic Mn–ZnS/MOF/MIP Fluorescent Sensor for Highly Sensitive and Selective Sulfathiazole Detection
Source: Biosensors (Basel). 2026 Aug 24;16(9):459. doi: 10.3390/bios16090459 (PMC13604594; doi:10.3390/bios16090459)
Supplement: Supplementary file 1 [file biosensors-16-00459-s001.zip › biosensors-4444376-supplementary.pdf]

SUPPLEMENTARY INFORMATION

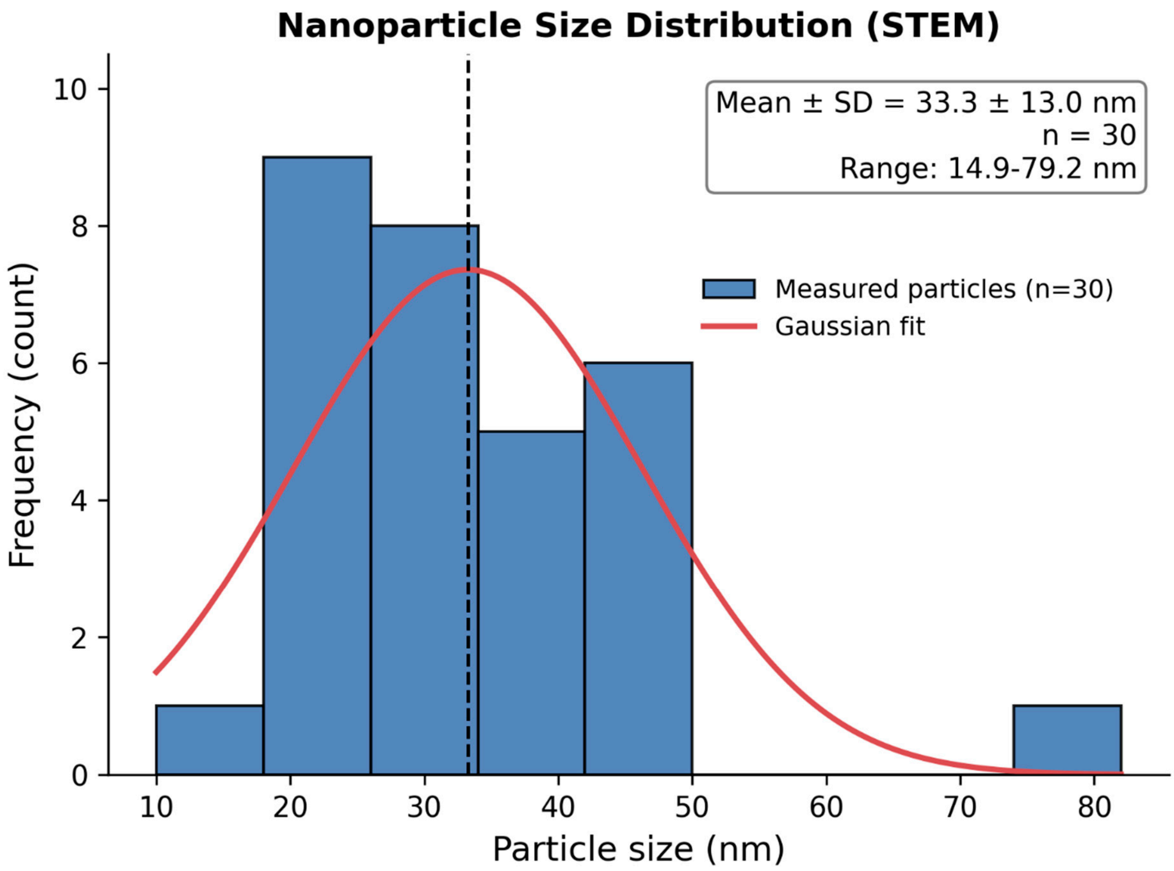

Figure S1. Size distribution histogram of Mn-ZnS quantum dots determined from STEM image analysis

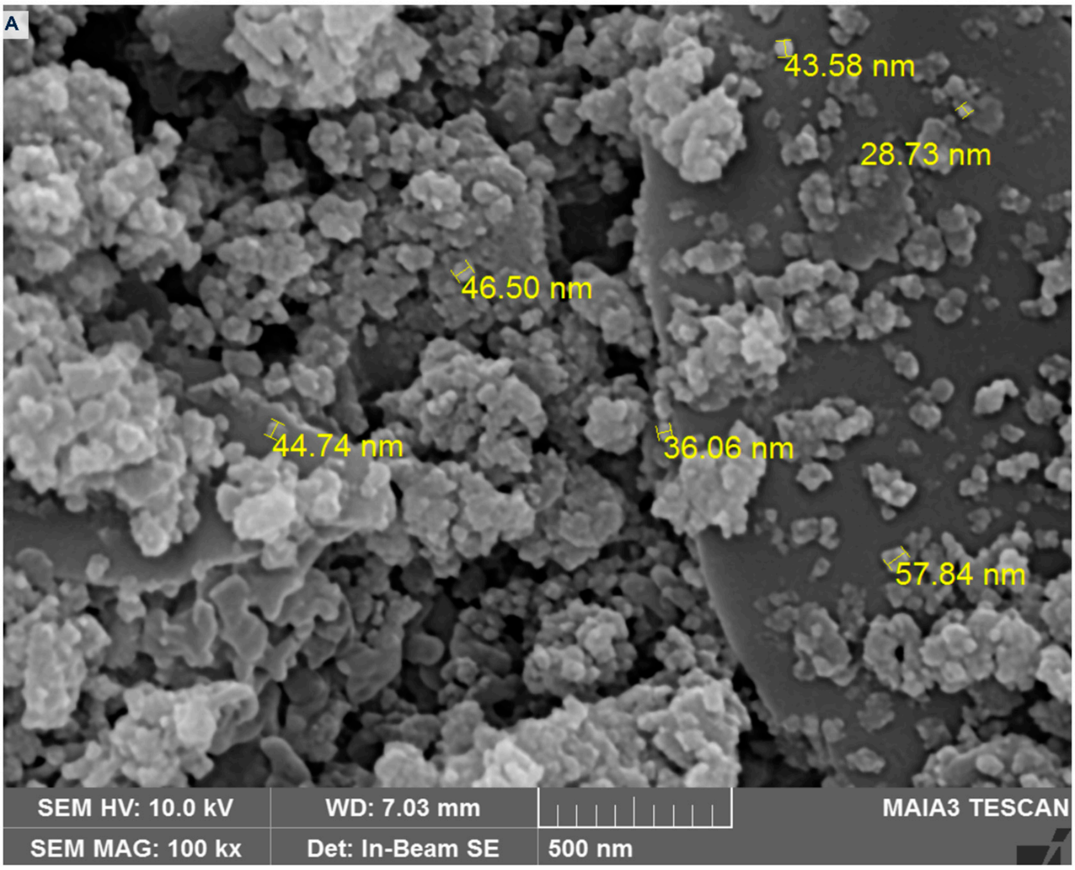

Figure S2: SEM image of Mn-ZnS-MOF

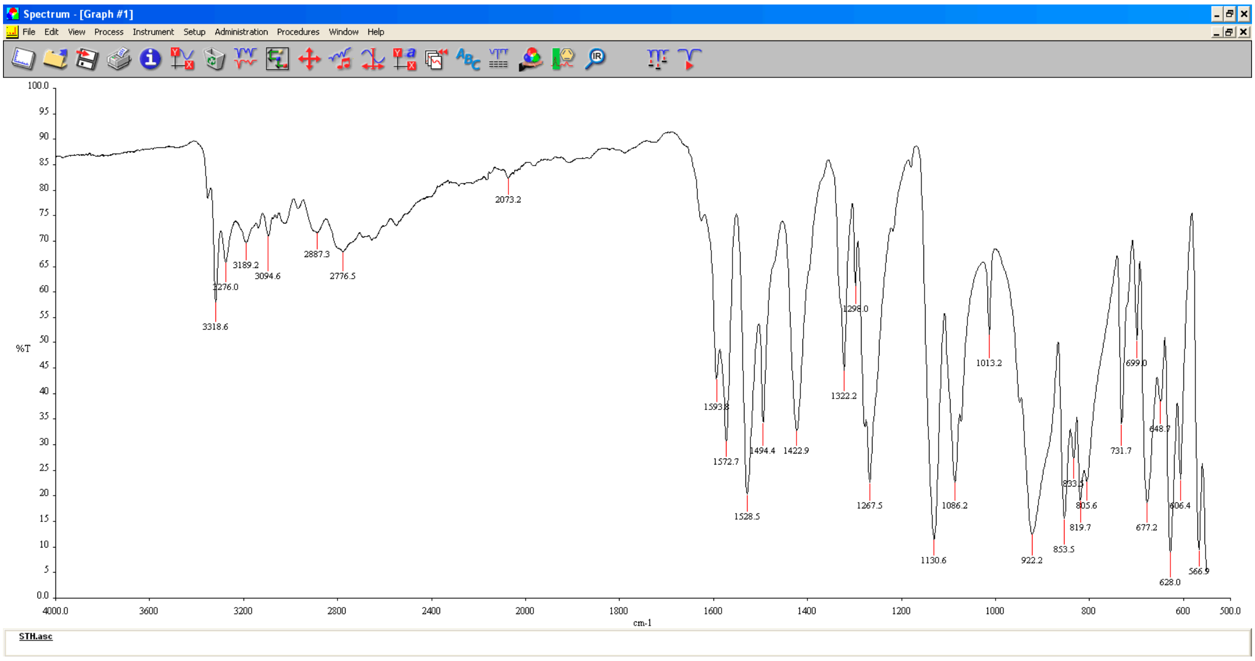

Figure S3. The FTIR spectrum of STH.

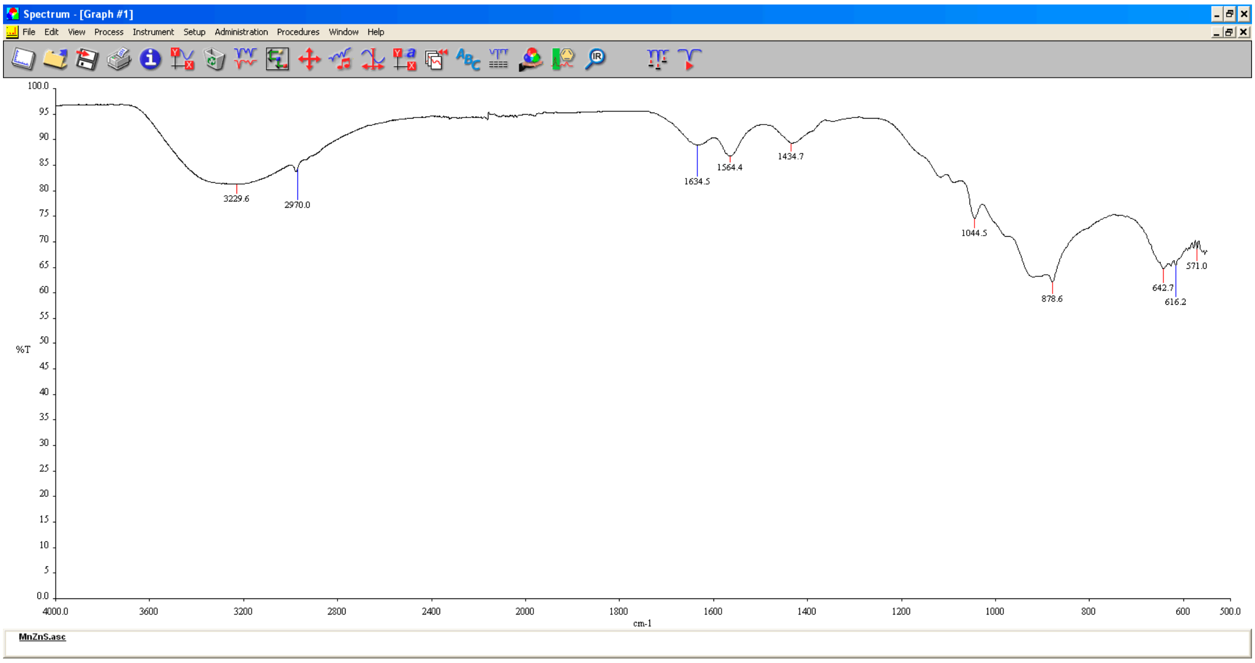

Figure S4. The FTIR spectrum of Mn-ZnS.

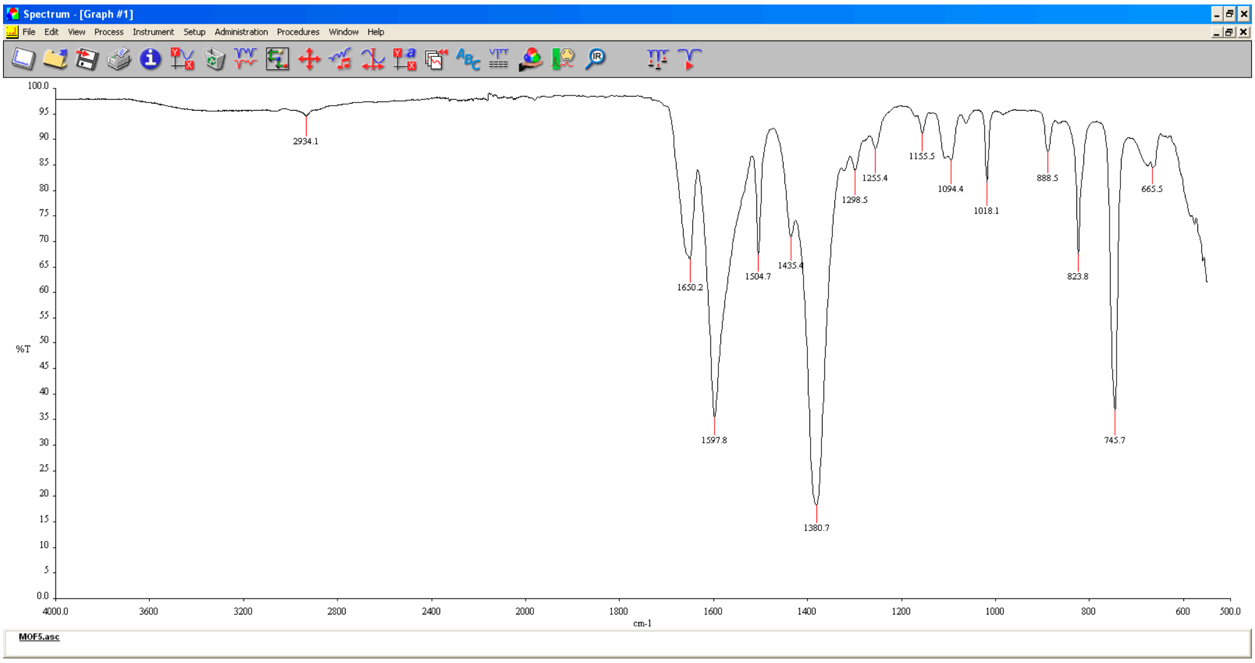

Figure S5. The FTIR spectrum of MOF5.

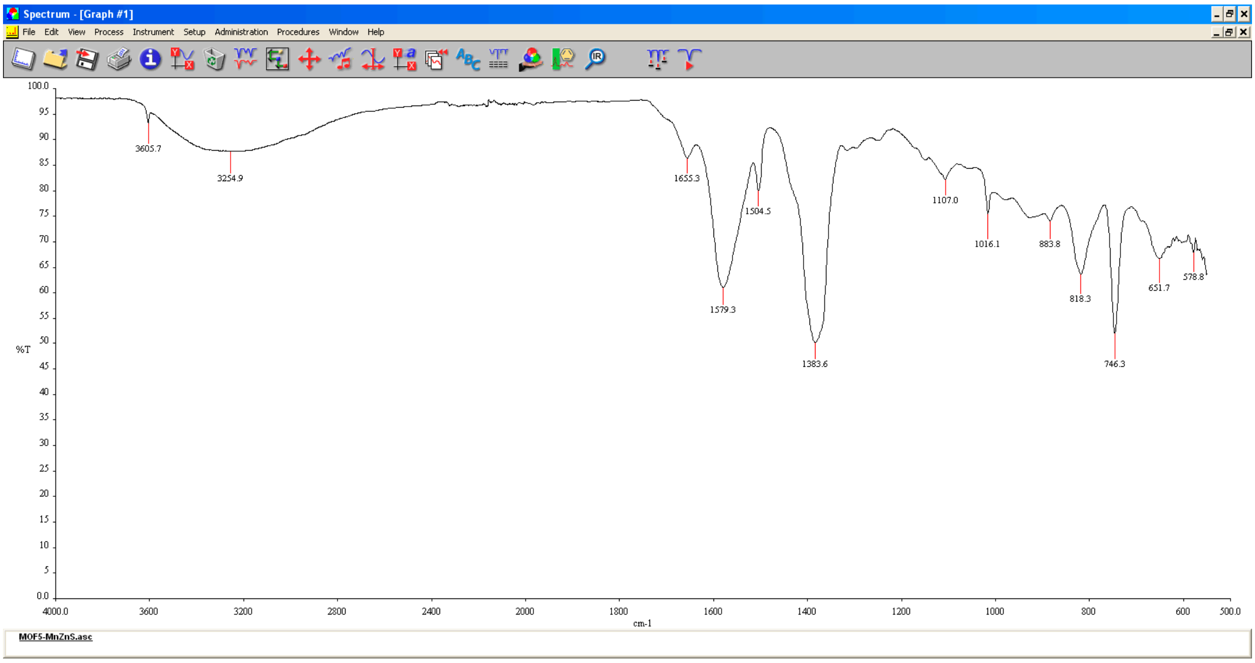

Figure S6. The FTIR spectrum of MOF-Mn-ZnS.

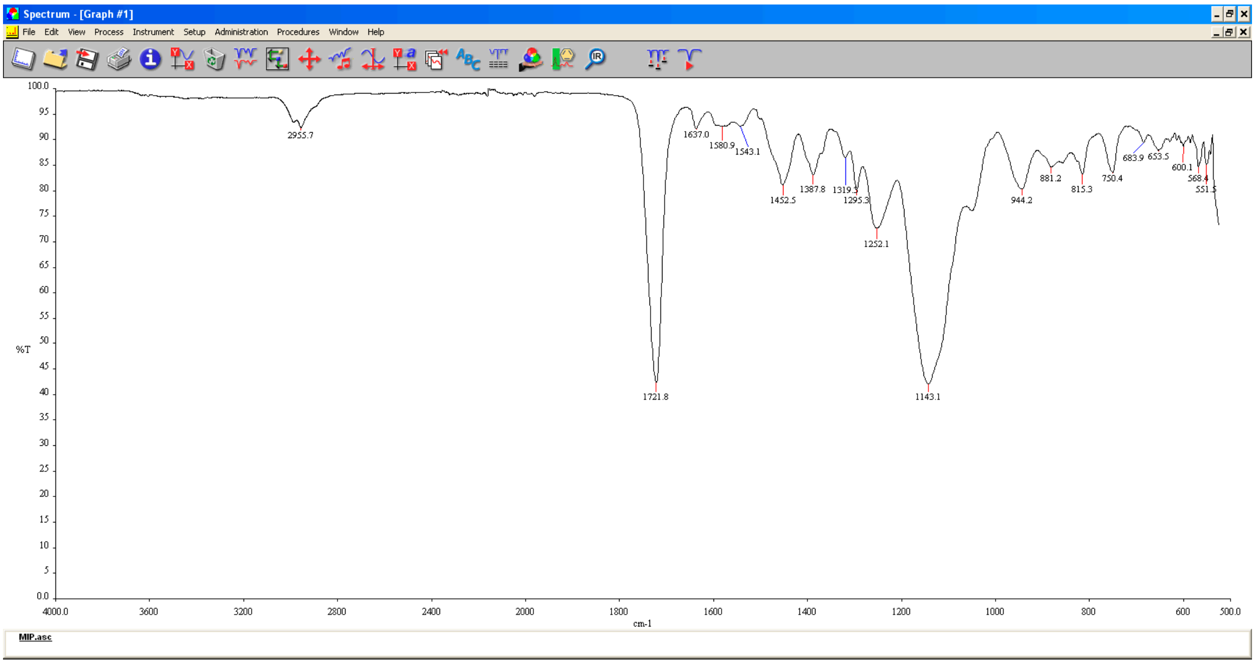

Figure S7. The FTIR spectrum of Mn-ZnS-MOF5-MIP before removal of STH.

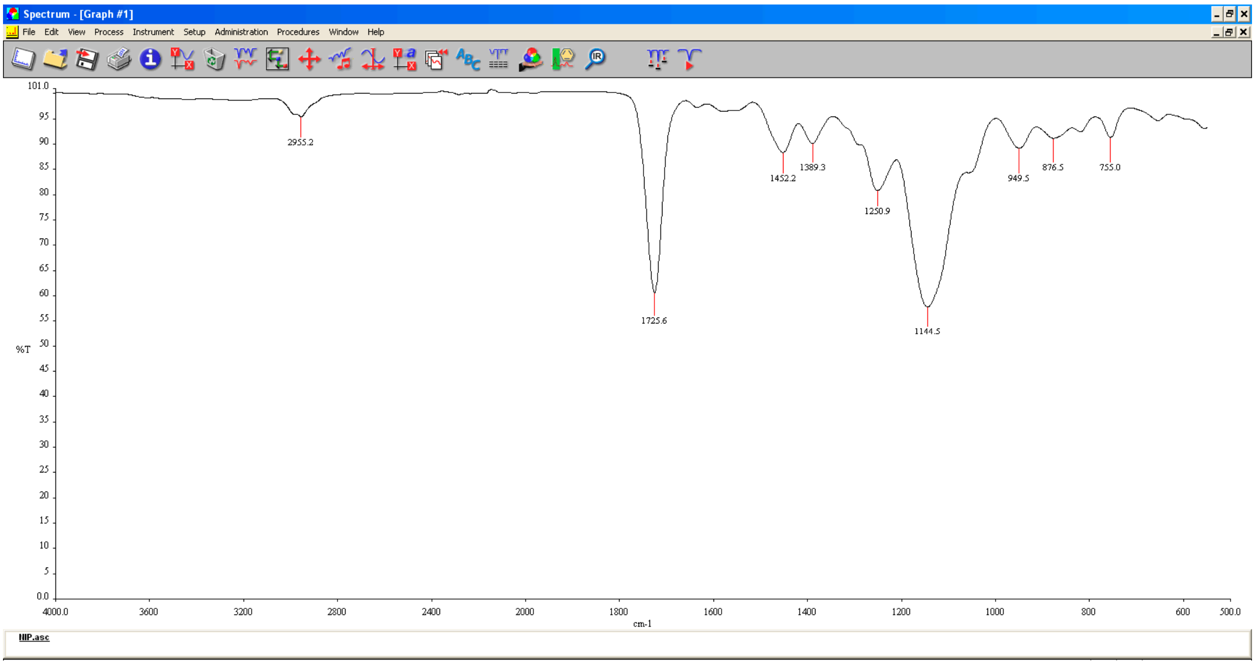

Figure S8. The FTIR spectrum of Mn-ZnS-MOF5-NIP

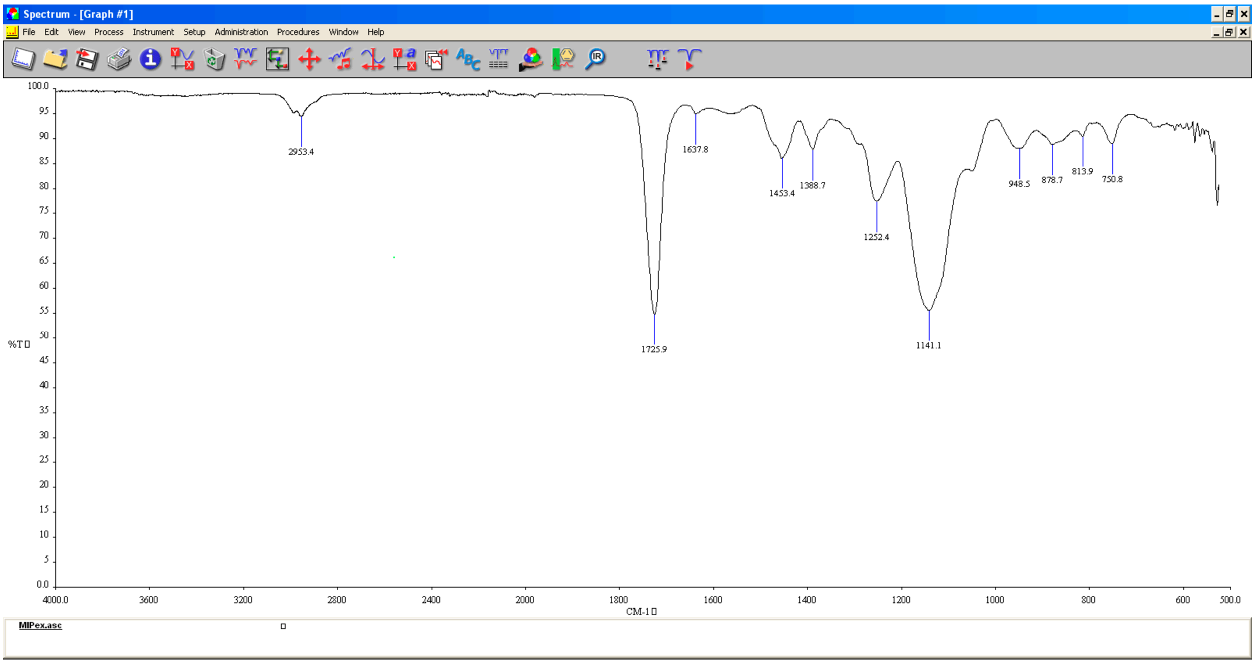

Figure S9. The FTIR spectrum of Mn-ZnS-MOF5-MIP after removal of STH.

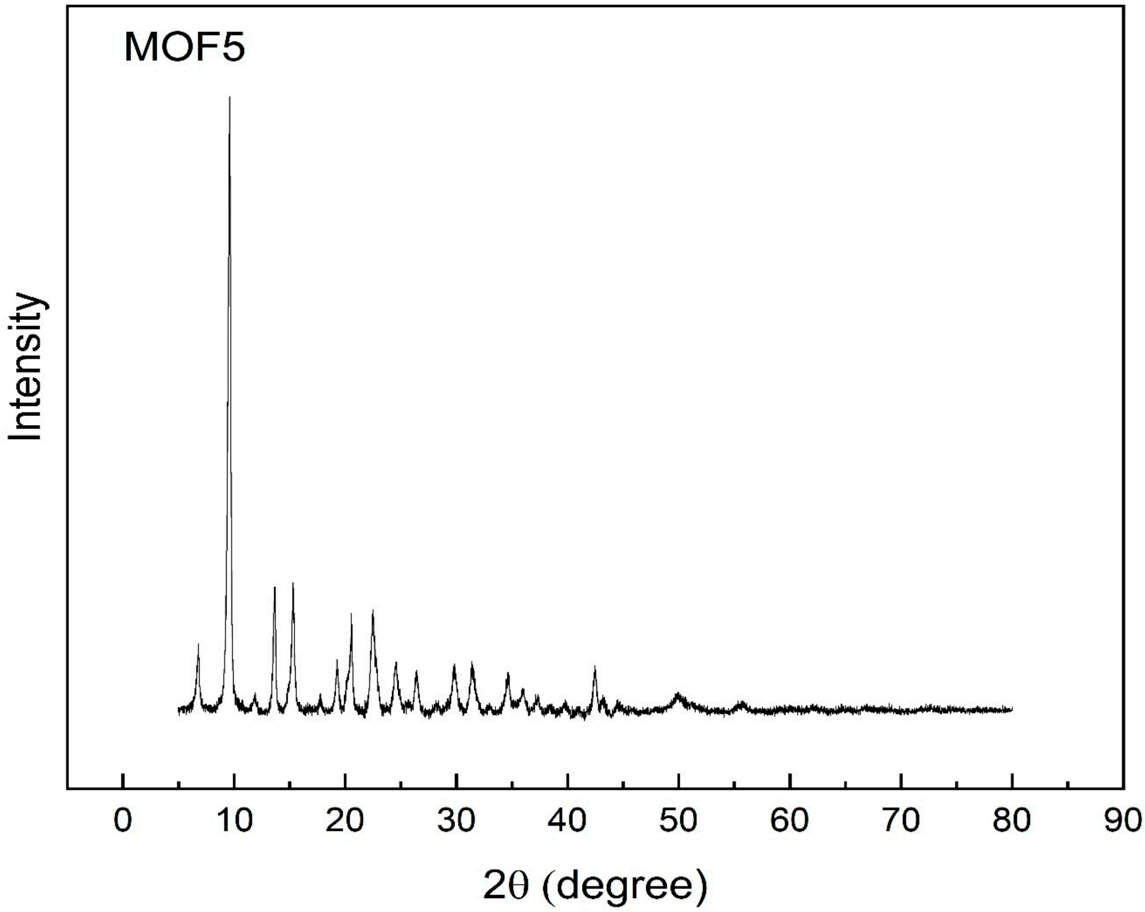

Figure S10. XRD pattern of MOF5

To verify the phase identity of the pristine metal–organic framework employed in this study, the powder XRD pattern of as-synthesized MOF-5 was collected separately and compared with simulated and experimental patterns reported in the literature (Figure S10;[1-3]). The pattern exhibits the diagnostic low-angle reflections at $2\theta \approx 6.8°$, 9.7°, 13.7°, and 15.4°, characteristic of a highly crystalline, non-interpenetrated cubic MOF-5 framework [2]. Slight variations in relative peak intensities occasionally observed across MOF-5 samples in the literature have been attributed to partial pore occupancy by residual $Zn(OH)_2$ or solvent molecules, or to framework interpenetration, rather than to the presence of impurity phases [1]. These results confirm that the crystalline phase incorporated into the Mn-ZnS-MOF composite (Fig. 2b) corresponds to authentic MOF-5 rather than an amorphous or secondary zinc-oxo species.

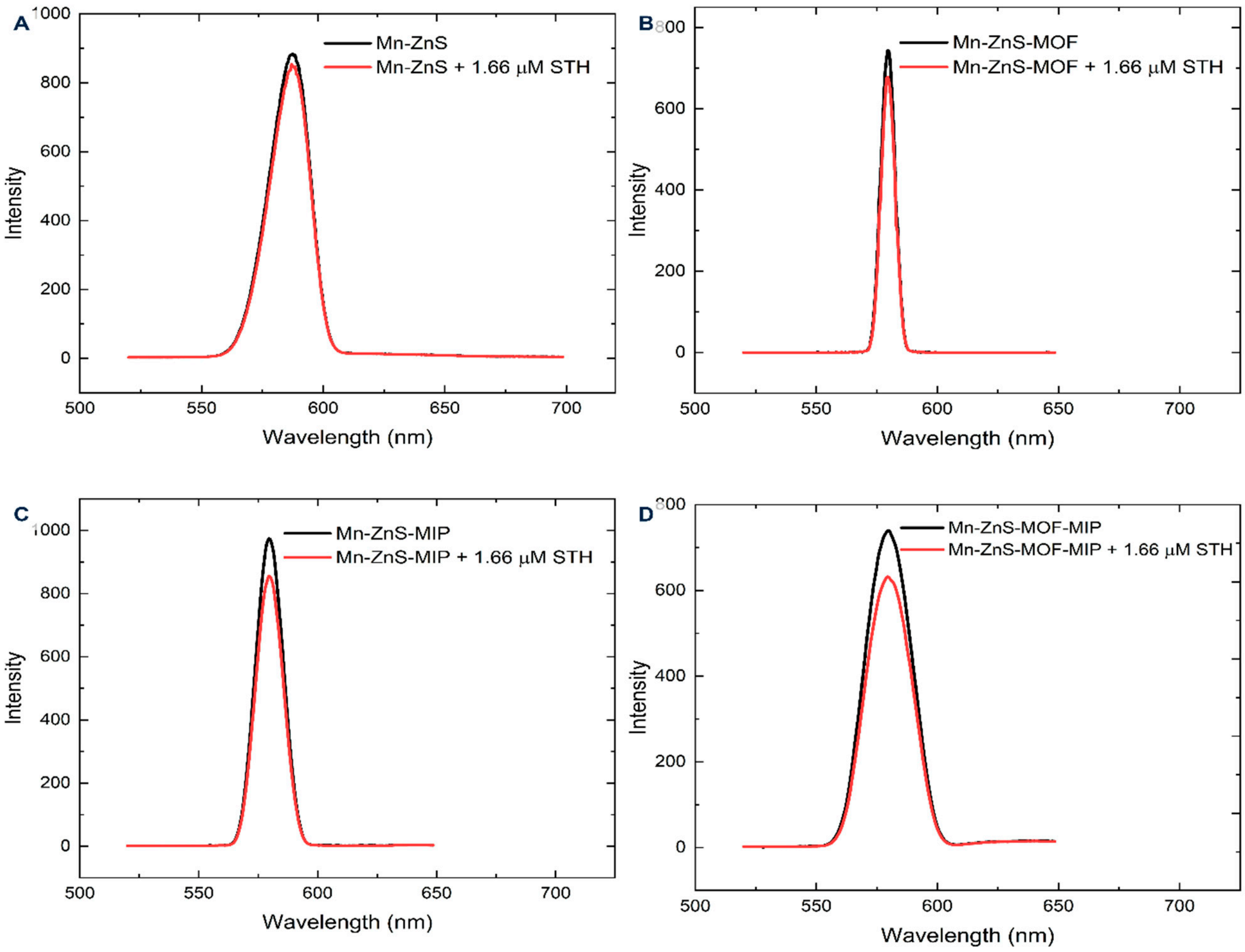

Figure S11. Fluorescence emission spectra of (A) Mn-ZnS, (B) Mn-ZnS-MOF, (C) Mn-ZnS-MIP, and (D) Mn-ZnS-MOF-MIP before (black) and after (red) the addition of 1.66 μM STH.

Temperature-Dependent Stern–Volmer Analysis

To further evaluate the fluorescence quenching mechanism, Stern–Volmer measurements were performed at three temperatures (25, 35, and 45 °C). The 25 °C dataset corresponds to the standard sensing measurement performed under ambient laboratory conditions using the optimized 1:1 monomer: template ratio material that is already presented in Fig.6b. For the 35 °C and 45 °C measurements, working solutions of Mn-ZnS-MOF-MIP at each STH concentration (0–11.5 μM) were equilibrated in a thermostated water bath set to the target temperature for at least 15 min prior to measurement; solution temperature was verified using a calibrated thermometer immediately before and after each fluorescence reading to confirm it remained within ±1 °C of the target value. Samples were transferred to the LS-55 Fluorescence Spectrometer and measured immediately, with excitation at 290 nm.

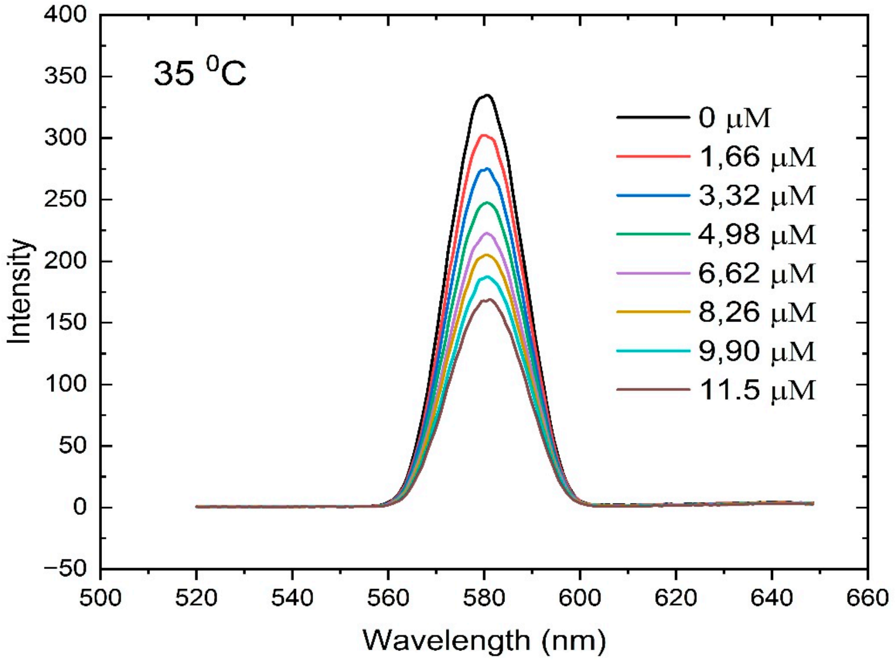

Figure S12. Fluorescence emission spectra of Mn-ZnS-MOF-MIP in the presence of increasing STH concentrations (0–11.5 μM) recorded at 35 °C .

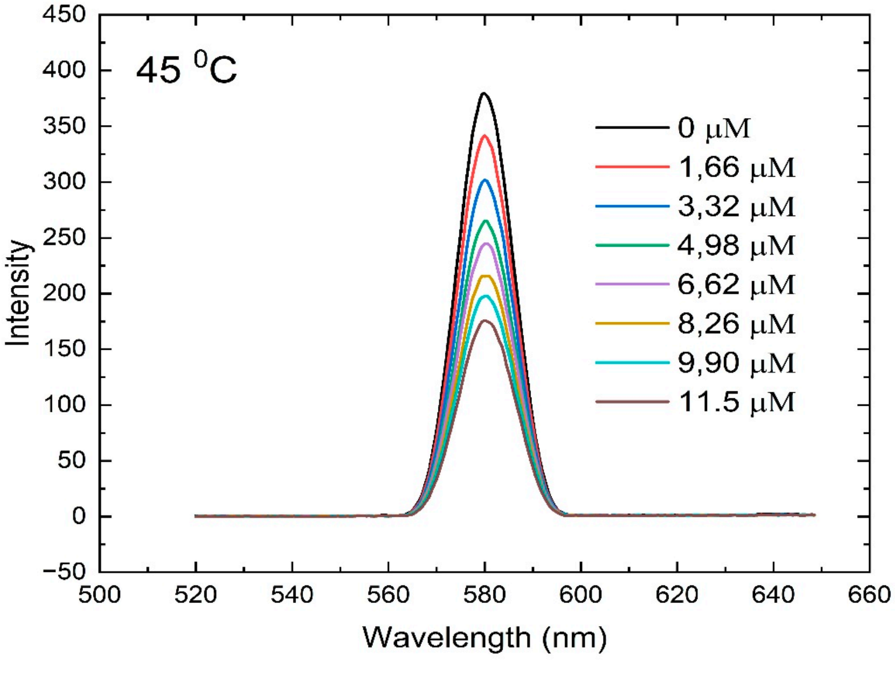

Figure S13. Fluorescence emission spectra of Mn-ZnS-MOF-MIP in the presence of increasing STH concentrations (0–11.5 μM) recorded at 45 °C .

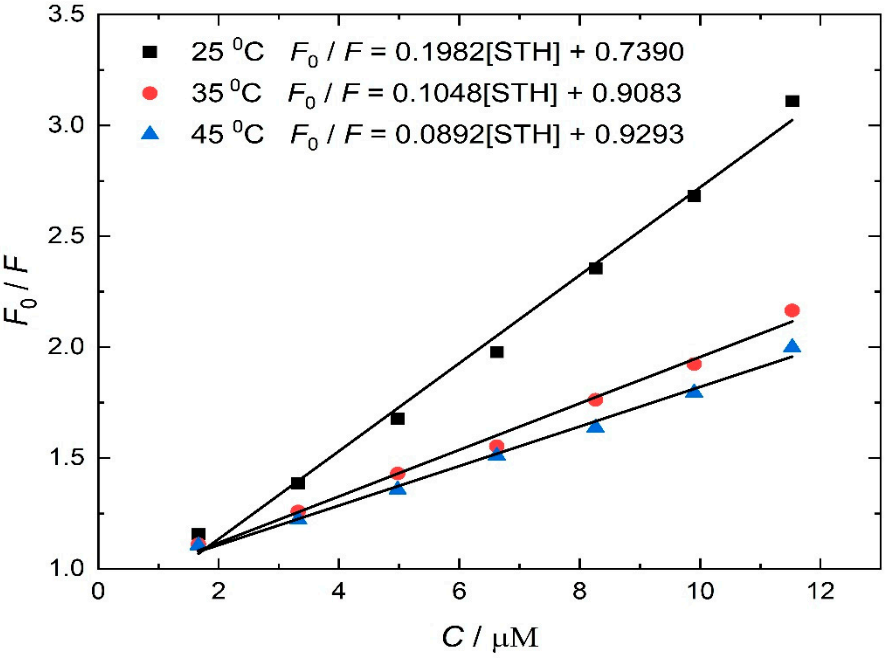

Figure S14. Temperature-dependent Stern–Volmer plots for Mn-ZnS-MOF-MIP at 25, 35, and 45 °C.

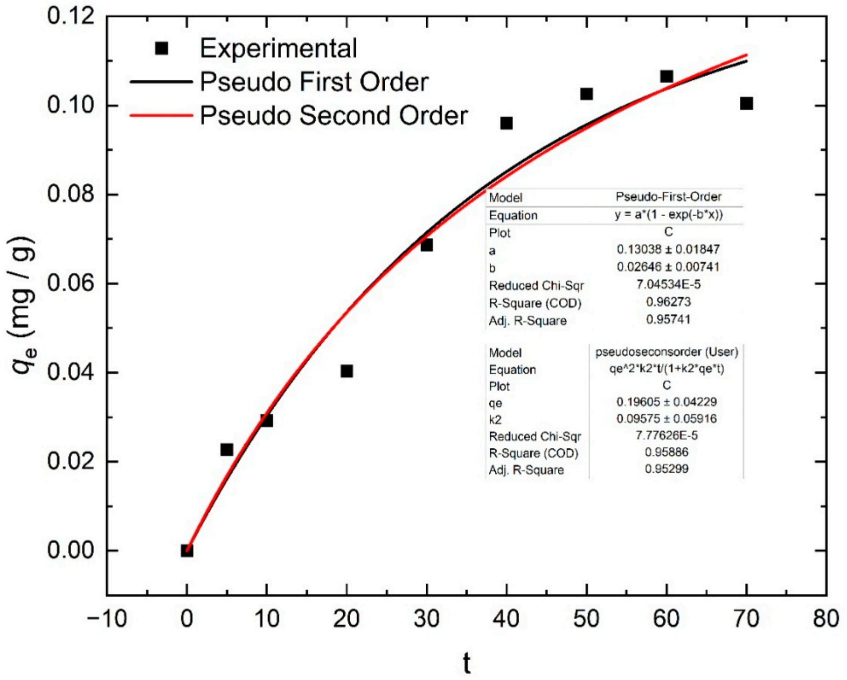

Figure S15. The experimental kinetics for the STH adsorption on Mn-ZnS-MOF-NIP and experimental data fitted to the pseudo-first-order and pseudo-second-order model.

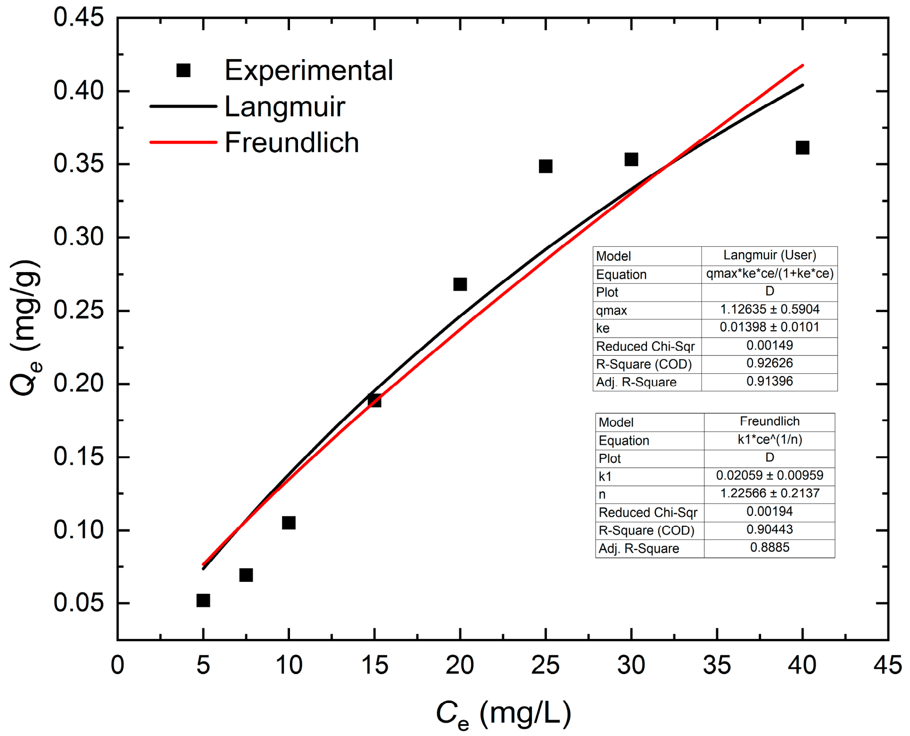

Figure S16. Experimental and fitted Langmuir and Freundlich isotherms for STH adsorption onto Mn-ZnS-MOF-NIP

The Long-term Stability

The long-term stability of the sensor to the prepared STH concentration level was determined periodically as it was stored under defined conditions (4°C). As seen in Fig. S14 that the sensor maintained about 80.71% of the original intensity of the sensing signal. This only showed a negligible change and had no systematic pattern in order to affirm the high stability of the imprinted polymer film against chemical and physical changes. This also showed that the binding pockets remained unchanged. Overall, the combination of reproducibility and stability results proved that the designed MIP sensor not only has high selectivity and sensitivity but also basic qualities needed to make it a reliable analytical device, with the low RSD ensuring uniformity in batches, as well as excellent stability in storage, which indicates good potential for the device's practical applications.

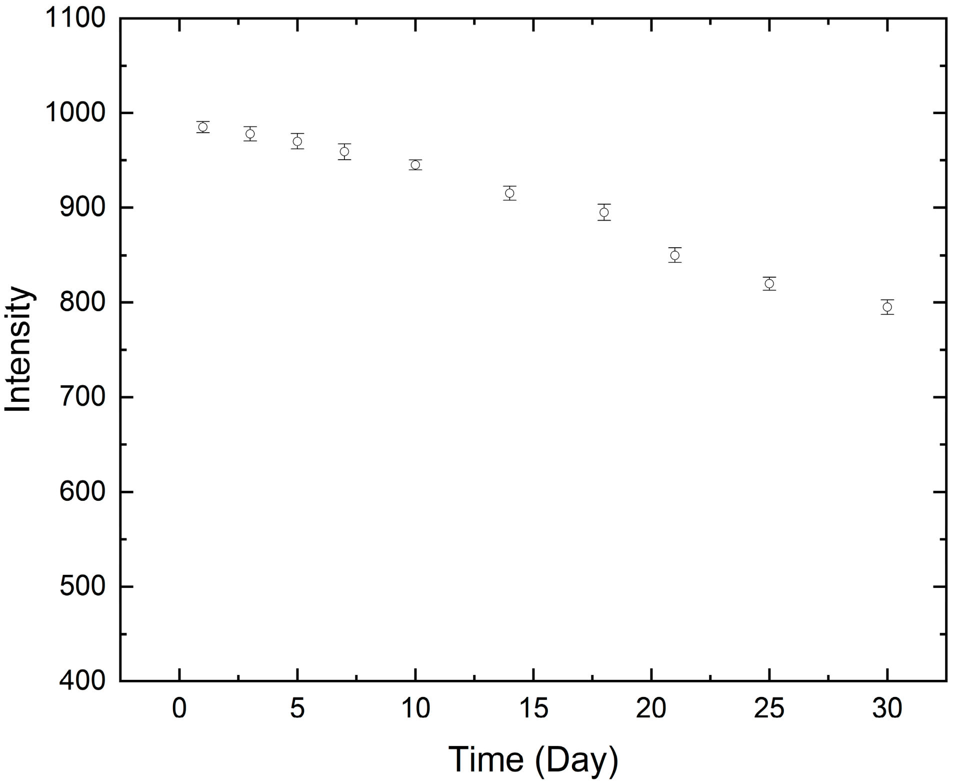

Figure S17. The long-term stability of the fluorescent probe.

Regeneration Studies

Regeneration of the developed sensing platform was evaluated over five consecutive binding–regeneration cycles using a single sensor throughout. All incubation, regeneration, and rinsing steps were carried out at room temperature, and after each step (binding, wash, and rinse) the sensor material was isolated by centrifugation (5000 rpm, 5 min) and filtered through a 0.22 μm membrane filter to remove aggregates and ensure a homogeneous dispersion prior to fluorescence measurement. In each cycle, the sensor was incubated with 5 μM STH for 10 min under the optimized conditions (pH 7, aqueous medium), and the fluorescence intensity was recorded . The sensor was then regenerated by washing with methanol/acetic acid (4:1, v/v) for 20 min under stirring, rinsed extensively with deionized water, and re-equilibrated in fresh PBS for 10 minutes; fluorescence intensity was measured again at this point (F_0), prior to the next binding step. Quenching efficiency for each cycle was calculated as $(F_0 - F)/F_0 \times 100$. As shown in Figure S18, quenching efficiency remained considerably stable over the five cycles with no progressive decline in response, indicating that the sensor retains its imprinted-cavity integrity and rebinding capacity with reuse.

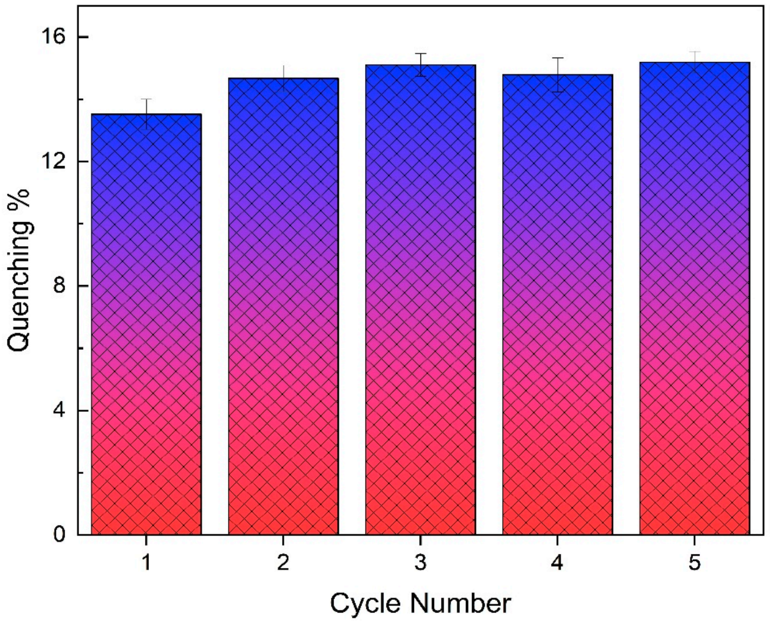

Figure S18. Reusability of the Mn-ZnS-MOF-5-MIP sensor for STH detection over repeated regeneration cycle.

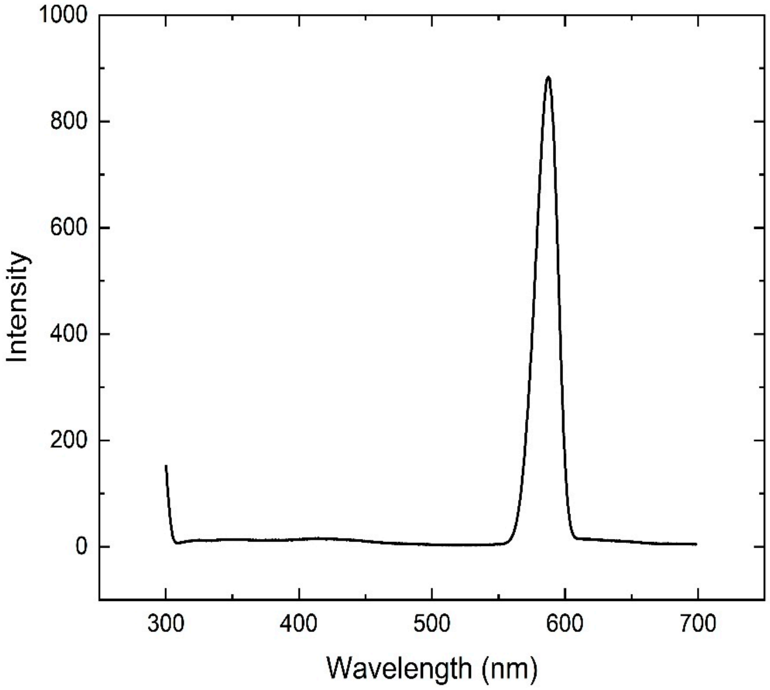

Figure S19. Fluorescence emission spectrum of the Mn-ZnS/MOF/MIP

Table S1. Comparison of the analytical performance of the Mn–ZnS/MOF/MIP fluorescent sensor with previously reported methods for the determination of sulfathiazole (STH).

Method / Platform	Technique	Linear Range	LOD	Reference
N,B,F co-doped carbon dots	Fluorescence	0.008–10 µg / mL	21.6 nM	[4]
Cu nanoclusters + ionic-liquid membrane microextraction	Fluorometric	0.75–100 µmol/L	110 nM	[5]
CdTe quantum dots @ MIP	Fluorescence	10–60 µg/kg	2.31 nM	[6]
Magnetic MIP + microextraction	HPLC–UV	0.25-4 ng/ml	5.1 nM	[7]
SPE + immunoaffinity cleanup	HPLC–UV	50-1000 µg/kg	97.9 nM	[8]
Ti_3AlC_2@$SmVO_4$ heterojunction/GCE	Electrochemical	0.01–364 µM	5.8 nM	[9]
Triadic Mn–ZnS/MOF/MIP	Fluorescence	1.6 to 19.6 µM	13.75 nM	This work

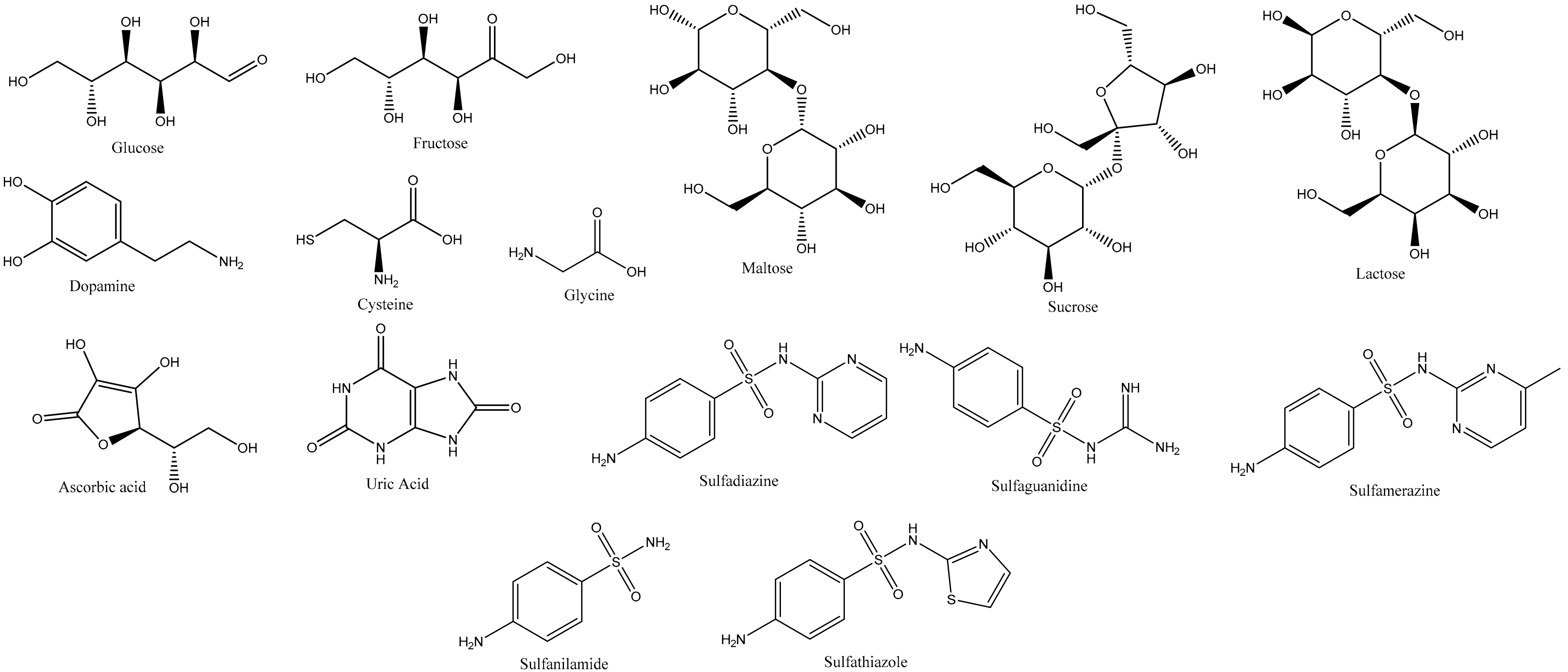

Chart S1. Chemical structures of the compounds evaluated in selectivity study corresponding to Figure 6h.

References

[1] J. Hafizovic, M. Bjørgen, U. Olsbye, P.D.C. Dietzel, S. Bordiga, C. Prestipino, C. Lamberti, K.P. Lillerud, The Inconsistency in Adsorption Properties and Powder XRD Data of MOF-5 Is Rationalized by Framework Interpenetration and the Presence of Organic and Inorganic Species in the Nanocavities, Journal of the American Chemical Society, 129 (2007) 3612-3620.
[2] S. Hausdorf, J.r. Wagler, R. Moβig, F.O.R.L. Mertens, Proton and Water Activity-Controlled Structure Formation in Zinc Carboxylate-Based Metal Organic Frameworks, The Journal of Physical Chemistry A, 112 (2008) 7567-7576.
[3] H. Li, M. Eddaoudi, M. O'Keeffe, O.M. Yaghi, Design and synthesis of an exceptionally stable and highly porous metal-organic framework, Nature, 402 (1999) 276-279.

[4] L. Chen, Y. Liu, G. Cheng, Z. Fan, J. Yuan, S. He, G. Zhu, A novel fluorescent probe based on N, B, F co-doped carbon dots for highly selective and sensitive determination of sulfathiazole, Science of The Total Environment, 759 (2021) 143432.

[5] S. Sadeghi, S. Oliaei, Microextraction of sulfathiazole from milk and honey samples using a polymeric ionic liquid membrane followed by fluorometric determination, Journal of Food Composition and Analysis, 97 (2021) 103774.
[6] B. Mortari, A. Wong, S. Khan, R.F. Dutra, M. Del Pilar Taboada Sotomayor, Advanced Fluorometric Detection of Sulfathiazole Antibiotics in Food Samples with Molecularly Imprinted Polymer Coated CdTe Quantum Dots, ACS Omega, 10 (2025) 33910-33920.
[7] R. Karami-Osboo, R. Miri, K. Javidnia, M.H. Shojaee, F. Kobarfard, Extraction and determination of sulfadiazine and sulfathiazole in milk using magnetic solid phase extraction-HPLC-UV, Analytical Methods, 7 (2015) 1586-1589.
[8] A. Zotou, C. Vasiliadou, Selective Determination of Sulfonamide Residues in Honey by SPE-RP-LC with UV Detection, Chromatographia, 64 (2006) 307-311.
[9] S. Maheshwaran, R. Balaji, S.-M. Chen, Y.-C. Liao, N. Chandrasekar, S. Ethiraj, M.S. Samuel, Fabrication of 2D–0D Ti3AlC2@SmVO4 heterojunction nanocomposites for ultrasensitive electrochemical detection of sulfathiazole in environmental samples, Journal of Environmental Chemical Engineering, 10 (2022) 108956.
